# Hepatitis B Virus X Protein Function Requires Zinc Binding

**DOI:** 10.1128/JVI.00250-19

**Published:** 2019-07-30

**Authors:** Dhivya Ramakrishnan, Weimei Xing, Rudolf K. Beran, Saketh Chemuru, Henry Rohrs, Anita Niedziela-Majka, Bruno Marchand, Upasana Mehra, Aleš Zábranský, Michal Doležal, Martin Hubálek, Iva Pichová, Michael L. Gross, Hyock Joo Kwon, Simon P. Fletcher

**Affiliations:** a Gilead Sciences, Inc., Foster City, California, USA; b Department of Chemistry, Washington University, St. Louis, Missouri, USA; c Institute of Organic Chemistry and Biochemistry of the Czech Academy of Sciences, Prague, Czech Republic; University of Southern California

**Keywords:** DDB1, HBx, hepatitis B virus, Smc5/6 complex

## Abstract

The host structural maintenance of chromosomes 5/6 complex (Smc5/6) suppresses hepatitis B virus (HBV) transcription. HBV counters this restriction by expressing the X protein (HBx), which redirects the cellular DNA damage-binding protein 1 (DDB1)-containing E3 ubiquitin ligase to target Smc5/6 for degradation. However, the details of how HBx modulates the interaction between DDB1 and Smc5/6 remain to be determined. In this study, we performed biophysical analyses of recombinant HBx and functional analysis of HBx mutants in HBV-infected primary human hepatocytes (PHH) to identify key regions and residues that are required for HBx function. We determined that recombinant HBx is soluble and exhibits stoichiometric zinc binding when expressed in the presence of DDB1. Mass spectrometry-based hydrogen-deuterium exchange and cysteine-specific chemical footprinting of the HBx:DDB1 complex identified several HBx cysteine residues (located between amino acids 61 and 137) that are likely involved in zinc binding. These cysteine residues did not form disulfide bonds in HBx expressed in human cells. In line with the biophysical data, functional analysis demonstrated that HBx amino acids 45 to 140 are required for Smc6 degradation and HBV transcription in PHH. Furthermore, site-directed mutagenesis determined that C61, C69, C137, and H139 are necessary for HBx function, although they are likely not essential for DDB1 binding. This CCCH motif is highly conserved in HBV as well as in the X proteins from various mammalian hepadnaviruses. Collectively, our data indicate that the essential HBx cysteine and histidine residues form a zinc-binding motif that is required for HBx function.

**IMPORTANCE** The structural maintenance of chromosomes 5/6 complex (Smc5/6) is a host restriction factor that suppresses HBV transcription. HBV counters this restriction by expressing HBV X protein (HBx), which redirects a host ubiquitin ligase to target Smc5/6 for degradation. Despite this recent advance in understanding HBx function, the key regions and residues of HBx required for Smc5/6 degradation have not been determined. In the present study, we performed biochemical, biophysical, and cell-based analyses of HBx. By doing so, we mapped the minimal functional region of HBx and identified a highly conserved CCCH motif in HBx that is likely responsible for coordinating zinc and is essential for HBx function. We also developed a method to produce soluble recombinant HBx protein that likely adopts a physiologically relevant conformation. Collectively, this study provides new insights into the HBx structure-function relationship and suggests a new approach for structural studies of this enigmatic viral regulatory protein.

## Abstract (precis)

The structural maintenance of chromosomes 5/6 complex (Smc5/6) is a host restriction factor that suppresses HBV transcription. HBV counters this restriction by expressing HBV X protein (HBx), which redirects a host ubiquitin ligase to target Smc5/6 for degradation. Despite this recent advance in understanding HBx function, the key regions and residues of HBx required for Smc5/6 degradation have not been determined. In the present study, we performed biochemical, biophysical, and cell-based analyses of HBx. By doing so, we mapped the minimal functional region of HBx and identified a highly conserved CCCH motif in HBx that is likely responsible for coordinating zinc and is essential for HBx function. We also developed a method to produce soluble recombinant HBx protein that likely adopts a physiologically relevant conformation. Collectively, this study provides new insights into the HBx structure-function relationship and suggests a new approach for structural studies of this enigmatic viral regulatory protein.

## INTRODUCTION

Approximately 240 million individuals worldwide are chronically infected with hepatitis B virus (HBV), and over 650,000 people die per year owing to HBV-associated liver diseases such as cirrhosis and hepatocellular carcinoma (HCC) (1, 2). Several nucleos(t)ide inhibitors, as well as interferon alpha (IFN-α), are approved for the treatment of chronic hepatitis B (CHB). However, because these therapies rarely lead to functional cure, there is an urgent need to develop new antiviral therapies.

HBV X protein (HBx) is a 154-amino-acid protein expressed shortly after infection, which localizes predominantly to the nucleus of HBV-infected primary human hepatocytes (PHH) (3). HBx is an attractive therapeutic target for the treatment of CHB because this viral protein is required for the maintenance of the covalently closed circular DNA (cccDNA) HBV genome in a transcriptionally active state (4–6). Recent studies demonstrated that cccDNA transcription is inhibited by the structural maintenance of chromosomes 5/6 complex (Smc5/6) and that HBx redirects a cellular DNA damage-binding protein 1 (DDB1)-containing E3 ubiquitin ligase to target this complex for degradation (7, 8). This newly defined mechanism also accounts for the ability of HBx to transactivate episomal DNA templates (7, 9).

Various studies have identified key regions required for HBx function, although these results have not been validated in the context of a natural HBV infection system. One such study identified a conserved alpha-helical motif in HBx called the H-box (residues 89 to 98), which is the minimal region required for DDB1 binding (10–12). In addition, transactivation- and plasmid-based HBV replication studies suggested that HBx_58–140_ (residues 58 to 140) and HBx_43–154_, respectively, constitute the minimal functional region of HBx (13, 14). Interestingly, the central and C-terminal regions of HBx contain a number of highly conserved cysteine (C) and histidine (H) residues, suggesting that coordination of metal ions and/or formation of disulfide bonds may be important for HBx function. A recent study described the elemental characterization of recombinant HBx which was fused to maltose-binding protein (MBP) to enable expression in a soluble form (15). This demonstrated that the MBP-HBx fusion contains iron and zinc ions but did not establish the functional relevance of this observation. Interestingly, zinc binding is essential for the function of a number of viral and host proteins that bind DDB1 (DCAFs [DDB1-Cul4-associated factors]). For example, the host cereblon protein, the Vpx protein of HIV-2, and the V protein of paramyxovirus SV5 (SV5-V) coordinate either one or two zinc ions via CCCC, CCHH, and/or CCCH motifs (16–18). Zinc binding plays a critical structural role in each of these proteins. In contrast to these other DCAFs, the structure of HBx has not been determined, and it is not known whether zinc (or other metal ion) binding is required for HBx function.

In the present study, we performed biophysical analyses of recombinant HBx (in both the presence and absence of DDB1), as well as *in vitro* HBV infection studies in PHH, to (i) determine whether HBx function requires metal ion binding and (ii) identify key regions and residues required for HBx function.

## RESULTS

### Recombinant HBx is soluble and exhibits stoichiometric zinc binding when expressed in the presence of DDB1.

We first expressed and purified a series of recombinant proteins to characterize metal ion binding by HBx in the presence and absence of DDB1 (Table 1). HBx and SV5-V (another viral DCAF) were expressed in Escherichia coli, and DDB1 was expressed and purified from insect cells. HBx, SV5-V, and DDB1 were purified using various affinity columns followed by size exclusion chromatography (SEC). HBx was also coexpressed with DDB1 or expressed as a fusion with DDB1 in insect cells and purified using tag affinity columns, a MonoQ column, and SEC. The design of the fusion protein was based upon a construct that was used to identify Smc5/6 as an HBx interaction partner (7). By generating a fusion protein, we were able to form an HBx:DDB1 complex of defined stoichiometry that was stable and could be purified to homogeneity. Purification tags were removed from the proteins during the purification process, with the exception of HBx, which contained an N-terminal His tag. The purified proteins were mostly monomeric (or formed a 1:1 complex in the case of coexpressed HBx:DDB1) and monodisperse by analytical ultracentrifugation (AUC) and dynamic light scattering (DLS) (Table 1). Strikingly, in contrast to HBx expressed alone, HBx expressed in the presence of DDB1 was soluble in the absence of detergent. To our knowledge, this represents the first time that full-length HBx protein has been successfully expressed and purified in a soluble form in the absence of detergents or fusion to a physiologically irrelevant protein (e.g., MBP).

**TABLE 1:** Biophysical characterization of HBx, DDB1, and HBx-DDB1 complexes^*a*^

Protein(s)	Species determined by AUC (%)		%Pd by DLS	Mean molar ratio by ICP-MS ± SEM^*c*^		Detergent required for solubility
	Main species^*b*^	Other species		Zn/protein	Fe/protein	
DDB1	6.4S (99)	10.9S (1)	9	≤0.2 ± 0.1^*f*^	≤0.1 ± 0.1^*f*^	No
HBx	1.6S (80)	3.5S (7)	ND	0.0 ± 0.0^*f*^	0.0 ± 0.0^*f*^	Yes^*d*^
HBx:DDB1 coexpressed	6.6S (99)	9.9S (1)	12	0.7 ± 0.1	≤0.1 ± 0.1^*f*^	No
HBx-DDB1 fusion	6.8S (92.4)	10.4S (6.7)	19	1.3 ± 0.2	≤0.1 ± 0.0^*f*^	No
HBx_CCCH_-DDB1 fusion^*e*^	ND	ND	ND	0.3 ± 0.0	≤0.1 ± 0.0^*f*^	No
SV5-V	2.1S (94.7)	5.2S (2)	18	1.9 ± 0.1	0.0 ± 0.0^*f*^	No

a AUC, analytical ultracentrifugation; DLS, dynamic light scattering; %Pd, percent polydispersity; ND, not determined.

b The main species was a monomer of each protein or a one-to-one complex in the case of the HBx:DDB1 coexpressed construct.

c ICP-MS (inductively coupled plasma mass spectrometry) data are expressed as mean molar ratios ± SEM (*n* = 2 to 7 independent experiments).

d HBx expressed alone required 0.01% Fos-choline 14 for solubilization.

e HBx_CCCH_ refers to the HBx C61A/C69A/C137A/H139A mutant protein.

f Values represent the limit of quantitation.

**FIG 1:**
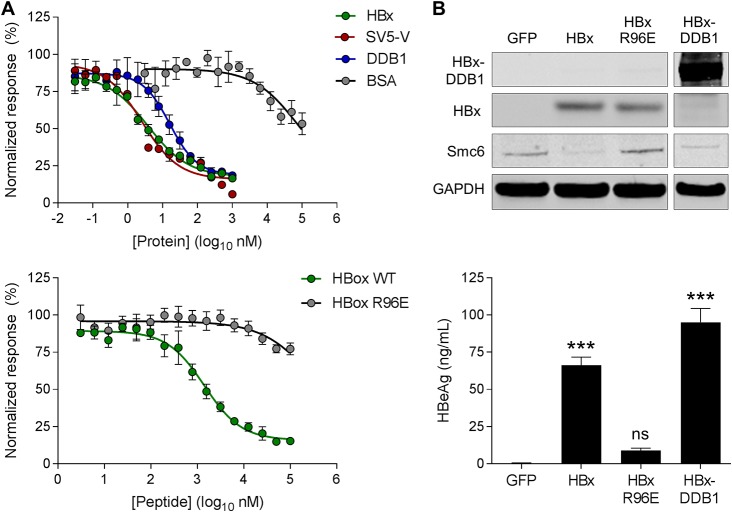
Characterization of recombinant HBx proteins. (A, top) TR-FRET analysis of the coexpressed His-HBx:GST-DDB1 complex in the presence of recombinant untagged HBx, SV5-V, DDB1, and BSA. (Bottom) TR-FRET analysis of the coexpressed His-HBx:GST-DDB1 complex in the presence of the wild-type (WT) HBx H-box peptide and a mutant HBx R96E H-box peptide. (B, top) Western blot analysis of PHH lysates collected 72 h after transduction with lentiviruses expressing GFP, Myc-HBx, Myc-HBx R96E, or the Myc-HBx-DDB1 fusion. HBx and the HBx-DDB1 fusion were detected using an anti-Myc antibody. Data are representative of results from 3 independent experiments. (Bottom) PHH were transduced with the same lentiviruses and 1 day later were infected with HBVΔX. Extracellular HBeAg levels at day 13 postinfection are expressed as a percentage of the value for the HBx control; the bar height indicates the mean of data from 3 independent experiments, and the error bars represent the SEM. Statistical significance relative to the GFP control was calculated by one-way ANOVA with Dunnett’s multiple-comparison correction. ***, *P* < 0.001; ns, not significant (*P* > 0.05).

We next characterized the recombinant HBx proteins by performing a series of DDB1 binding studies using time-resolved fluorescence resonance energy transfer (TR-FRET). For these studies, His-HBx and glutathione *S*-transferase (GST)-DDB1 were expressed separately or coexpressed and then purified using affinity columns, ion exchange, and SEC. We first demonstrated that His-HBx expressed alone (detergent solubilized) could bind GST-DDB1 (data not shown). We then confirmed that the His-HBx:GST-DDB1 interaction for both His-HBx expressed alone as well as the coexpressed His-HBx:GST-DDB1 complex could be disrupted by the addition of recombinant untagged HBx, SV5-V, or DDB1 but not bovine serum albumin (BSA) (Table 2 and Fig. 1A). We also verified that a wild-type HBx H-box peptide (HBx_88–102_) disrupted the His-HBx:GST-DDB1 interaction (albeit less efficiently than full-length HBx protein), whereas an H-box peptide containing a mutation that reduces DDB1 binding (R96E) did not (Table 2 and Fig. 1A). These TR-FRET data demonstrate that recombinant HBx (expressed alone or coexpressed with DDB1) interacts with DDB1 in a manner that can be competitively inhibited.

**TABLE 2:** Competitive inhibition of the HBx:DDB1 interaction^*a*^

Competitor^*b*^	Mean IC_50_ (nM) (SEM)	
	His-HBx and GST-DDB1 expressed separately^*c*^	His-HBx:GST-DDB1 coexpressed
HBx	61 (±5)	9 (±5)
SV5-V	7 (±2)	4 (±2)
DDB1	204 (±25)	16 (±0.2)
HBx H-box peptide (wild type)	5,000 (±1,000)	1,560 (±93)
HBx H-box peptide (R96E mutant)	166,000 (±37,000)	>100,000
BSA	>3,000	>100,000

a The interaction between His-HBx and GST-DDB1 was measured by TR-FRET. Data are expressed as means (SEM) (*n* = 2 to 5 independent experiments). Note that the wild-type SV5-V H-box peptide also competitively inhibited HBx:DDB1 binding (data not shown).

b Competitor proteins and peptides were not tagged. BSA, bovine serum albumin.

c HBx expressed alone required detergent for solubilization.

Since it is not possible to perform comparable competitive binding studies with the recombinant HBx-DDB1 fusion protein, we confirmed the biological relevance of this construct in primary human hepatocyte (PHH) studies. We transduced PHH with lentiviruses expressing green fluorescent protein (GFP), HBx, DDB1-binding-deficient HBx (HBx R96E), or the HBx-DDB1 fusion. As expected, both HBx and the HBx-DDB1 fusion promoted the degradation of Smc6, whereas GFP and HBx R96E did not (Fig. 1B). We also evaluated the same lentiviruses in PHH infected with an HBx-negative virus (HBVΔX), using extracellular hepatitis B virus e antigen (HBeAg) levels as a surrogate marker of HBV transcription (19). An analogous HBx *trans*-complementation assay was recently used to evaluate the activity of X proteins from various mammalian hepadnaviruses (20). Consistent with the ability of the HBx constructs to mediate Smc6 degradation, HBVΔX transcription was rescued only by HBx and the HBx-DDB1 fusion (Fig. 1B). Collectively, these data demonstrate that the HBx-DDB1 fusion construct is functional in PHH, thereby suggesting that the recombinant HBx-DDB1 fusion protein can adopt a physiologically relevant structure.

We next used inductively coupled plasma mass spectrometry (ICP-MS) to determine whether the recombinant proteins contained iron or zinc ions (Table 1). Both coexpressed HBx:DDB1 as well as the HBx-DDB1 fusion protein contained zinc in an ∼1:1 molar ratio. Zinc was not detected in HBx expressed in the absence of DDB1. SV5-V contained two zinc ions, and DDB1 contained no zinc ions, consistent with previous structural studies (17). Iron was not detected in any of the recombinant proteins. Because zinc binding plays a critical structural role in various host and viral DCAFs, the absence of zinc in HBx expressed alone suggests that this protein does not completely adopt a physiologically relevant conformation in the absence of DDB1.

### Biophysical analyses of recombinant HBx indicate that C61, C69, C115, and C137 are involved in zinc binding.

**FIG 2:**
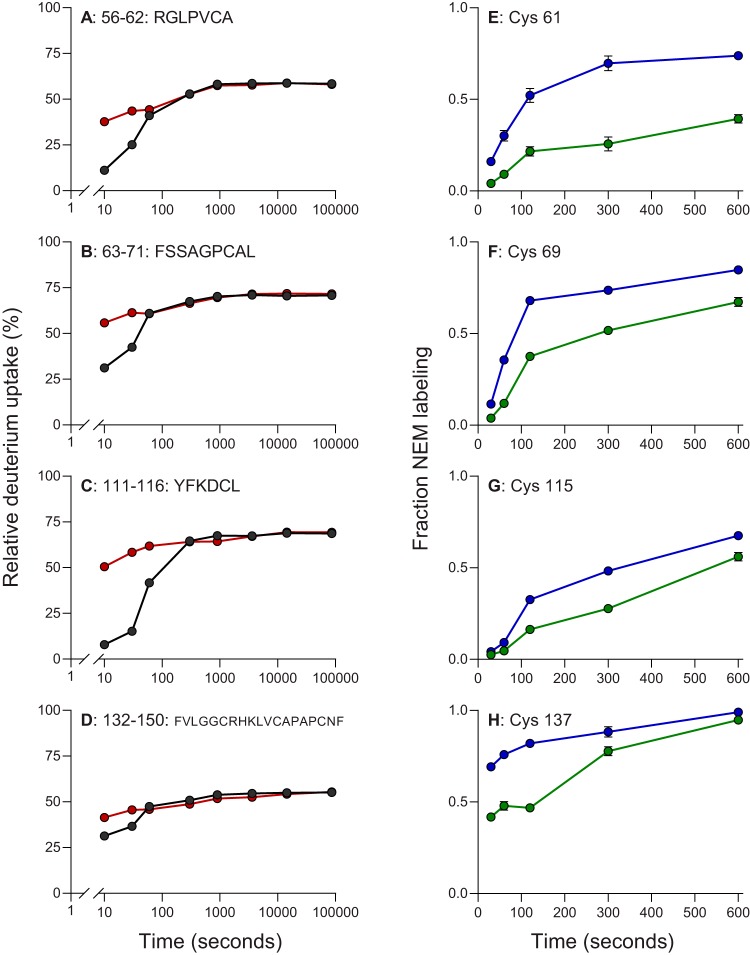
Identification of HBx cysteine residues protected upon zinc binding using HDX and kinetic NEM footprinting. (A to D) Comparison of deuterium uptake kinetic plots for select HBx peptides in the coexpressed HBx:DDB1 complex in the presence (black) and absence (red) of zinc. These peptides are representative of larger regions in the protein that show changes in HDX upon binding (as described in the text). (E to H) Kinetics of NEM labeling of select cysteine residues of HBx protein in the coexpressed HBx:DDB1 complex in the presence (green) and absence (blue) of zinc. The circles indicate the means, and the error bars represent the SEM. Statistical significance was calculated by a two-tailed *t* test. Only the four cysteines with significant differences (*P* < 0.01) are shown.

We next performed mass spectrometry-based hydrogen-deuterium exchange (HDX) analysis of the coexpressed HBx:DDB1 complex in the presence and absence of a zinc-chelating agent. The goals of these studies were to confirm that recombinant HBx binds zinc in the presence of DDB1 and to identify those regions of HBx that are involved in zinc binding. In this manner, we examined 28 overlapping peptides of HBx (95% coverage) and 194 peptides of DDB1 (89% coverage) (see Fig. S1 and S2 in the supplemental material). In line with the ICP-MS data, the HDX analysis indicated that HBx binds zinc when bound to DDB1. Almost all regions of the HBx protein (as represented by the peptic peptides) underwent rapid exchange, indicating that HBx has a highly exposed dynamic conformation. We found differences in deuterium uptake in some of the HBx peptides upon removal of zinc (Fig. 2A to D). The difference between the two states was mainly observed at early time points (10 to 60 s). By examining all peptides in those regions that showed changes in HDX upon zinc binding, we identified regions spanning positions 54 to 71, 86 to 102, 109 to 116, and 126 to 150 as being potentially involved in zinc binding. The involvement of these regions is either by direct zinc binding or by remote conformational changes induced by binding. Interestingly, the two states converge within 60 s of hydrogen-deuterium exchange, indicating that zinc association and dissociation are dynamic and that equilibrium likely has a significant off-rate. Of note, the HDX pattern of the HBx-DDB1 fusion protein in the presence and absence of a zinc-chelating agent was comparable to that of the coexpressed HBx:DDB1 complex (Fig. S3). In line with the solubility and zinc binding data, this suggests that the structure of HBx, when covalently tethered to DDB1, is similar to that of HBx in the coexpressed HBx:DDB1 complex.

In addition to locating various regions of HBx likely involved in zinc binding, HDX also provides insights into other sections of the proteins comprising the complex. In the absence of zinc, most of the regions in HBx undergo rapid uptake of deuterium (within 10 s, resembling a “burst phase”) to approximately 70% exchange, followed by a leveling off (Fig. S2). We attribute the leveling off to composite behavior over the region represented by the peptic peptide (i.e., exchange of both structured and unstructured sections). The leveling-off effect at <100% is not due to back-exchange, as there are regions in HBx and DDB1 that give ∼100% exchange. The overall HDX behavior indicates that HBx is largely unstructured, except for a few short regions (e.g., HBx_47–56_) that are structured and show little HDX. Another major difference is HBx_86–100_, which contains the H-box domain. This region becomes less dynamic in the presence of zinc binding, but its HDX resembles that of the rest of the protein. In contrast, most of the DDB1 regions show slower HDX uptake, with no “burst phase,” indicating that the protein is largely structured throughout (Fig. S1).

HDX defined various regions of HBx likely involved in zinc binding but did not identify specific residues that could play a role in binding this metal ion. We therefore used a cysteine-specific alkylating reagent, *N*-ethylmaleimide (NEM), to perform kinetic footprinting of zinc-bound and zinc-free HBx:DDB1 protein complexes. To induce differential labeling for cysteines that were buried or solvent exposed, we carried out time-dependent NEM labeling of the coexpressed HBx:DDB1 complex over a time range of 30 s to 10 min. The results obtained are consistent with the HBx regions identified in our HDX experiments, in that C61, C69, C115, and C137 showed significant decreases in NEM labeling between the zinc-free and zinc-bound states in the complex (Fig. 2E to H). None of the other cysteines in HBx or DDB1 showed significant NEM labeling differences. Thus, both HDX and NEM footprinting of cysteine residues indicated that four cysteines in HBx (C61, C69, C115, and C137) are involved in zinc binding by either serving as binding-site ligands or undergoing protein folding upon binding to become less solvent exposed.

### HBx cysteine residues do not form disulfide bonds.

Cysteine residues can also play an important role in the folding and stability of proteins by forming disulfide bonds. It was previously reported that recombinant HBx forms 4 to 5 disulfide bonds (21, 22). However, our mass spectrometry analysis of recombinant HBx expressed in the presence of DDB1 failed to identify any disulfide bonds (data not shown). We therefore evaluated whether any of the HBx cysteines could form disulfide bonds in a cellular context. Using a mass spectrometry-based proteomics approach, we mapped the formation of disulfide bonds in hemagglutinin (HA)-tagged HBx (HA-HBx) transiently expressed in Huh-7 cells (Table 3) and HEK293T cells (data not shown). All 10 HBx cysteine residues were detected in the reduced form in both cell types under both nonreducing and reducing conditions. These data therefore indicate that the cysteine residues of HBx expressed in human cell lines do not form either intramolecular or intermolecular disulfide bonds. The absence of disulfide bonds in HBx under these conditions is in accordance with the generally accepted principle that disulfide bonds usually form in the lumen of the endoplasmic reticulum rather than in the reducing environment of the cytosol (23).

**TABLE 3:** Characterization of HBx cysteine residues in Huh-7 cells^*a*^

Cysteine position(s)^*b*^	Sequence^*c*^	Peptide position(s)^*d*^	Confidence (%)^*e*^	MW (charge state)^*f*^
6, 7	LCCQLDPAR	2, 3	98.89	1,131.516 (2)
17, 26	DVLCLRPVGAESCGRPFSGSLGTLSSPSPSAVPTDHGAHLSLR	4, 13	99.00	4,444.202 (5)
61, 69	GLPVCAFSSAGPCALR	5, 13	99.00	1,661.802 (2)
115	DCLFKDWEELGEEIR	2	99.00	1,937.883 (3)
137	LKVFVLGGCR	9	98.33	1,147.654 (3)
143, 148	LVCAPAPCNFFTSA	3, 8	99.00	1,553.701 (2)

a Analysis performed by LC-MS.

b Position(s) of the cysteine in the HBx protein sequence.

c Cysteine residues are underlined.

d Position(s) of the cysteine in the peptide sequence modified by iodoacetamide.

e Confidence of identification evaluated by Protein Pilot.

f MW, molecular weight of the peptide with the modification on cysteines. The charge state of the ion detected by mass spectrometry is in parentheses.

### Residues 45 to 140 constitute the minimal functional region of HBx.

**FIG 3:**
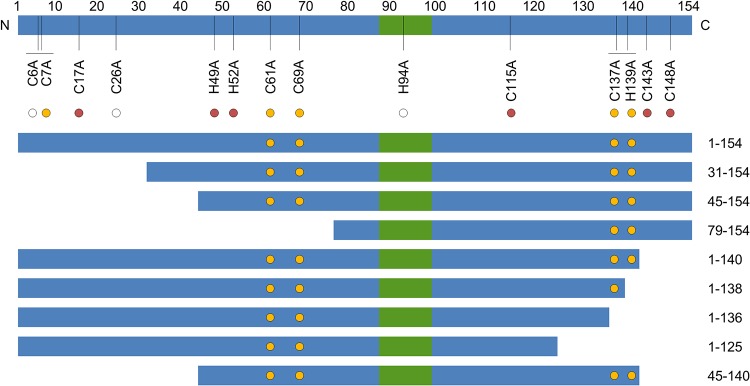
HBx mutants evaluated in PHH. Shown is a simplified schematic representation of the HBx protein; the green box denotes the H-box. HBx residue numbers are shown directly above the schematic, and HBx point mutations are displayed directly below. The circle color represents the degree of sequence conservation for each residue. The white circle denotes <90% sequence conservation in HBx. The red circle signifies >90% sequence conservation in HBx but not conservation in woodchuck, woolly monkey, and bat hepadnaviruses. The yellow circle denotes >90% sequence conservation in HBx and conservation in the woodchuck, woolly monkey, and bat hepadnaviruses. Truncation mutants are shown below the schematic, with the N and C termini of each mutant displayed on the right. The yellow circles in each HBx truncation mutant represent the positions (if present) of C61, C69, C137, and H139.

To complement the biophysical analysis of recombinant HBx, we next determined which regions and residues are essential for HBx function in PHH. In these studies, PHH were transduced with lentiviruses expressing GFP, full-length wild-type HBx (HBx_1–154_), or HBx with either a truncation or point mutation. The PHH were subsequently infected with HBVΔX, with HBx function being measured by determining intracellular Smc6 and extracellular HBeAg levels. The HBx mutants evaluated in this manner are summarized in Fig. 3. Of note, we previously demonstrated that lentivirus-expressed wild-type HBx predominantly localizes to the nucleus, which is consistent with the spatial distribution of HBx expressed in the context of natural infection (3).

**FIG 4:**
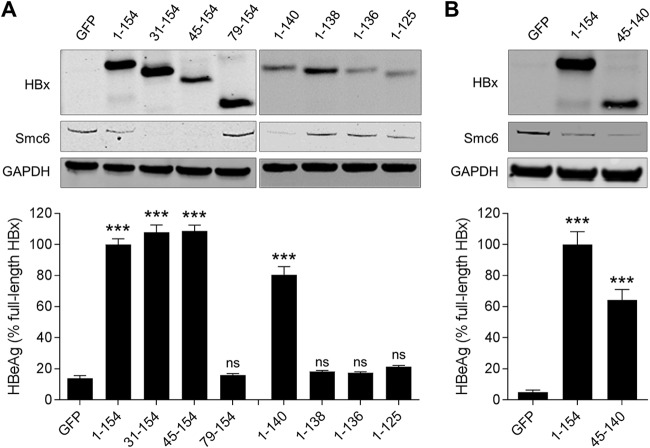
Identification of the regions required for HBx function in HBV-infected PHH. (A, top) PHH were transduced with lentiviruses expressing GFP, full-length HBx (HBx_1–154_), or HBx with either an N-terminal or C-terminal truncation. Cell lysates were analyzed by Western blotting at 72 h posttransduction. HBx was detected using an anti-Myc antibody. (Bottom) PHH were transduced with the same lentiviruses and 1 day later were infected with HBVΔX. Extracellular HBeAg was measured at day 13 postinfection. (B) PHH were transduced with lentiviruses expressing GFP, full-length HBx (HBx_1–154_), or HBx_45–140_. Cell lysates were analyzed by Western blotting at 72 h posttransduction. HBx was detected using an anti-Myc antibody. (Bottom) PHH were transduced with the same lentiviruses and 1 day later were infected with HBVΔX. Extracellular HBeAg was measured at day 13 postinfection. Data are expressed as a percentage of the value for the full-length HBx control; the bar height indicates the mean of data from 3 independent experiments, and the error bars represent the SEM. Statistical significance relative to the GFP control was calculated by one-way ANOVA with Dunnett’s multiple-comparison correction. ***, *P* < 0.001; ns, not significant (*P* > 0.05).

We first evaluated a series of N- and C-terminal HBx truncations. This revealed that HBx_31–154_, HBx_45–154_, and HBx_1–140_ promoted Smc6 degradation and significantly induced HBVΔX transcription, whereas HBx_79–154_, HBx_1–138_, HBx_1–136,_ and HBx_1–125_ did not (Fig. 4A). Collectively, these data indicate the HBx_45–140_ is the minimal functional region of HBx, and this was confirmed by demonstrating the functional activity of this truncation mutant (Fig. 4B). Overall, the functionality of the HBx truncations is consistent with the putative zinc-binding cysteines (C61, C69, C115, and C137) being potentially required for HBx function in PHH.

### C61, C69, C137, and H139 are essential for HBx function in PHH.

**FIG 5:**
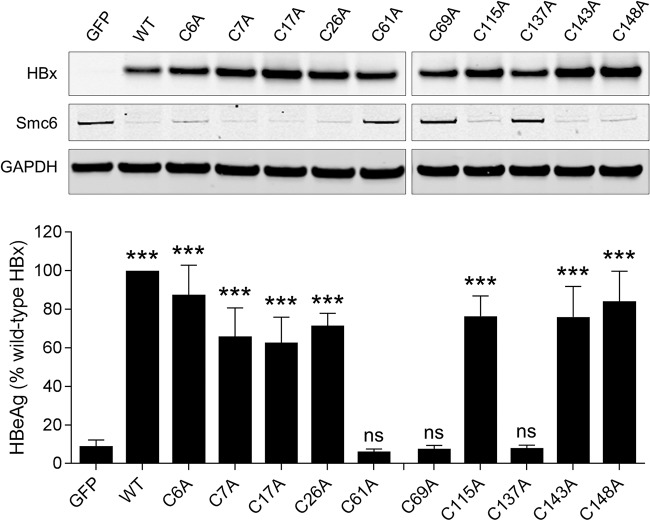
Identification of cysteine and histidine residues required for HBx function in HBV-infected PHH. (Top) PHH were transduced with lentiviruses expressing GFP, wild-type HBx (WT), or HBx mutants in which a single cysteine residue was mutated to alanine. Cell lysates were analyzed by Western blotting at 72 h posttransduction. HBx was detected using an anti-Myc antibody. (Bottom) PHH were transduced with the same lentiviruses and 1 day later were infected with HBVΔX. Extracellular HBeAg was measured at day 13 postinfection. Data are expressed as a percentage of the value for the wild-type HBx control; the bar height indicates the mean of data from 3 independent experiments, and the error bars represent the SEM. Statistical significance relative to the GFP control was calculated by one-way ANOVA with Dunnett’s multiple-comparison correction. ***, *P* < 0.001; ns, not significant (*P* > 0.05).

We next determined which cysteine residues are required for HBx function in PHH. Using the same experimental setup as the one described above, we evaluated HBx mutants in which cysteines were individually mutated to alanine (A) (Fig. 3). This demonstrated that C6A, C7A, C17A, C26A, C115A, C143A, and C148A induced Smc6 degradation and rescued HBVΔX transcription, whereas C61A, C69A, and C137A did not (Fig. 5). It is notable that C115 is not essential for HBx function in PHH despite being identified by NEM footprinting as being altered by zinc binding. A likely explanation is that HBx protein folding upon zinc binding leads to a conformational change around C115 that reduces solvent exposure and accessibility to alkylating agents.

**FIG 6:**
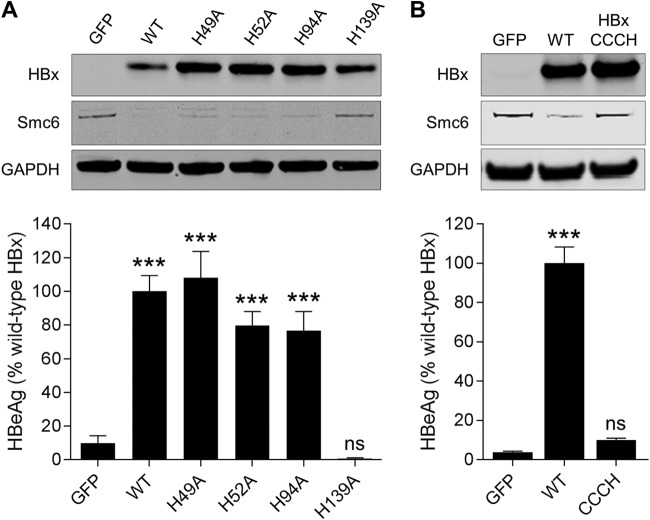
Identification of histidine residues required for HBx function in HBV-infected PHH. (A, top) PHH were transduced with lentiviruses expressing GFP, wild-type HBx (WT), or HBx mutants in which a single histidine residue was mutated to alanine. Cell lysates were analyzed by Western blotting at 72 h posttransduction. HBx was detected using an anti-Myc antibody. (Bottom) PHH were transduced with the same lentiviruses and 1 day later were infected with HBVΔX. Extracellular HBeAg was measured at day 13 postinfection. (B, top) PHH were transduced with lentiviruses expressing GFP, wild-type HBx, or HBx in which cysteine 61 (C61), C69, C137, and histidine 139 (H139) were all mutated to alanine (HBx_CCCH_). Cell lysates were analyzed by Western blotting at 72 h posttransduction. HBx was detected using an anti-Myc antibody. (Bottom) PHH were transduced with the same lentiviruses and 1 day later were infected with HBVΔX. Extracellular HBeAg was measured at day 13 postinfection. Data are expressed as a percentage of the value for the wild-type HBx control; the bar height indicates the mean of data from 2 independent experiments, and the error bars represent the SEM. Statistical significance relative to the GFP control was calculated by one-way ANOVA with Dunnett’s multiple-comparison correction. ***, *P* < 0.001; ns, not significant (*P* > 0.05).

Since zinc-binding motifs can comprise both cysteine and histidine residues, we also evaluated whether any histidine residues are essential for HBx function in PHH. The same alanine mutational approach demonstrated that H49A, H52A, and H94A promoted Smc6 degradation and significantly induced HBVΔX transcription, whereas H139A did not (Fig. 6A). Taken together with the biophysical analyses of recombinant HBx, these data suggest that HBx coordinates zinc via a CCCH motif comprising C61, C69, C137, and H139. In line with this interpretation, simultaneous mutation of these four residues (HBx_CCCH_) inhibited HBx function in PHH (Fig. 6B), and recombinant HBx_CCCH_ expressed as a fusion protein with DDB1 contained substantially less zinc than the HBx-DDB1 fusion protein (Table 1). In light of these data, we next evaluated whether various metal ions chelators [e.g., *N*,*N*,*N*′,*N*′-tetrakis(2-pyridinylmethyl)-1,2-ethanediamine) (TPEN)] that deplete intracellular zinc have an impact on HBx function in PHH. Unfortunately, these studies were unsuccessful owing to the well-characterized cytotoxic effect of these agents (24, 25).

Consistent with C61, C69, C137, and H139 being essential for HBx function, this CCCH motif is highly conserved in HBV (Fig. 3). Strikingly, the motif is also conserved in the X protein from woodchuck hepatitis virus (WHx) as well as the X proteins from various mammalian hepadnaviruses that can promote the degradation of Smc6 and functionally substitute for HBx in HBV-infected PHH (Fig. 3) (20). Therefore, zinc binding may also be required for the function of the X proteins from these mammalian hepadnaviruses.

### The HBx CCCH motif is not essential for DDB1 binding in PHH.

**FIG 7:**
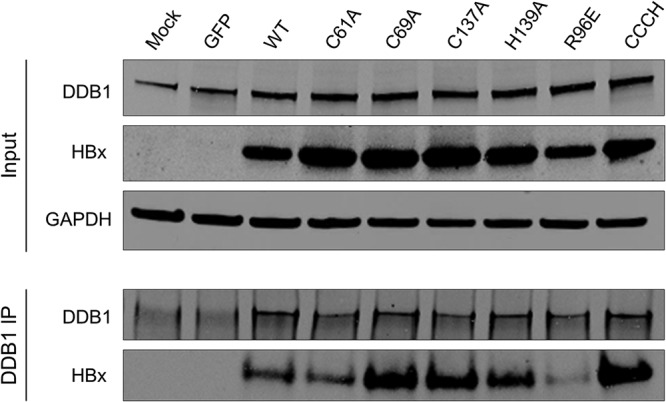
HBx C61, C69, C137, and H139 are not required for DDB1 binding. PHH were transduced with lentiviruses expressing the indicated proteins. (Top) Cell lysates were analyzed by Western blotting at 72 h posttransduction. (Bottom) DDB1 immunoprecipitation (IP) of the cell lysates was analyzed by Western blotting. HBx was detected using an anti-Myc antibody. Data are representative of results from 3 independent experiments.

Since zinc coordination plays a key structural role in other host and viral DCAFs, a potential consequence of mutation of the HBx CCCH motif is disruption of DDB1 binding. To evaluate this possibility, we transduced PHH with various HBx cysteine and histidine point mutants and performed immunoprecipitation (IP) of DDB1 followed by Western blot analysis of HBx. The R96E point mutant that reduces DDB1 binding was included as a control. As expected, wild-type HBx coimmunoprecipitated with DDB1, whereas only very low levels of the R96E mutant did so (Fig. 7). All the HBx cysteine and histidine mutants tested (including the CCCH quadruple mutant) coimmunoprecipitated with DDB1 (Fig. 7). These data suggest that C61, C69, C137, and H139 are not essential for HBx binding to DDB1 in PHH. This is consistent with the TR-FRET data that demonstrate that the HBx H-box peptide, as well as recombinant HBx expressed in the absence of DDB1 (which does not bind zinc), can bind DDB1 (Tables 1 and 2).

## DISCUSSION

The Smc5/6 complex was recently identified as an intrinsic restriction factor that is targeted for degradation by HBx protein (7, 8, 19). While this represents a significant advance in our understanding of HBx function, there is still much to learn about this viral regulatory protein. Based upon a series of biochemical, biophysical, and cell-based analyses, the present study indicates that highly conserved HBx cysteine and histidine residues (C61, C69, C137, and H139) form a zinc-binding motif that is required for HBx function. These data are consistent with a previous study that demonstrated that C61, C69, and C137 are required for HBx transactivation (13). Zinc binding plays a key structural role in a number of other viral DCAF proteins, including HIV-2 Vpx and SV5-V, as well as hosts DCAFs such as cereblon (16–18). Moreover, a number of viral proteins (e.g., HIV-1 Vif and herpes simplex virus [HSV] ICP0) that redirect other host ubiquitin ligases to target restriction factors for degradation are also dependent on zinc binding (26, 27).

Another notable finding from this study is that HBx is soluble and binds zinc when expressed in the presence of DDB1. This observation has two important implications. First, it suggests that DDB1 binding is required for HBx to adopt a physiologically relevant conformation. Second, it indicates that zinc binding is not essential for the interaction between HBx and DDB1. TR-FRET studies with recombinant HBx and DDB1 as well as coimmunoprecipitation (co-IP) experiments in PHH support this conclusion. The >80-fold difference in the 50% inhibitory concentrations (IC_50_s) of the HBx protein versus the HBx H-box peptide in the TR-FRET assay also suggests that the H-box is not the sole site of binding between HBx and DDB1. Consistent with our data, a yeast-two hybrid study reported that HBx_76–125_ is sufficient for DDB1 binding, indicating that the CCCH motif is not necessary for this interaction (12). In contrast, a second study reported that HBx C61 and C69 (and, by extension, zinc binding) are required for DDB1 binding (28). In light of these conflicting data, structural and biochemical studies of the DDB1-HBx-Smc5/6 ternary complex will be required to determine the precise role that zinc binding plays in HBx function.

Although the structure of the HBx H-box peptide binding to DDB1 has been determined (11), the structure of HBx protein has remained elusive. The present study provides promising new directions for HBx structural studies. First, it provides a method to produce soluble recombinant HBx protein that likely adopts a physiologically relevant conformation (see above). Second, consistent with previous studies, our mapping of the minimal function region of HBx demonstrates that the N-terminal region, which is thought to be unstructured (29), is not required for function. Future studies will therefore focus on determining the structure of N-terminally truncated HBx either coexpressed with DDB1 or expressed as a fusion with DDB1.

HBV can integrate into the host genome, typically via a double-stranded linear DNA form of the virus (30). Although HBV that integrates in this manner does not support viral replication, it can produce HBsAg and likely HBx. In this integrated form, the HBx protein is truncated by 3 or more amino acids (31–33). Previous studies have demonstrated that short C-terminal truncations do not impact the transactivation function of HBx (13, 32, 34–36). In line with these observations, we found that HBx with a C-terminal deletion of 14 amino acids (HBx_1–140_) promotes Smc6 degradation and *trans*-complements HBVΔX transcription. These data are consistent with the ability of X proteins from rodent and bat HBVs to substitute functionally for HBx in human cells despite the fact that they lack 9 to 12 of the C-terminal amino acids present in HBx (20). Because the C-terminal truncation removes the HBx stop codon, HBx-host cell fusion proteins can be produced by HBV integration events. The functionality of HBx fusion proteins was not the focus of the present study. However, a previous study demonstrated that an HBx-cell fusion protein derived from integrated HBV can retain transactivation activity (37), suggesting that HBx with a C-terminal extension with a host protein can be functional. Collectively, these data suggest that HBx produced from integrated HBV has the potential to promote the degradation of Smc5/6 and facilitate cccDNA transcription. This observation is of particular relevance to HBeAg-negative CHB, because integrated HBV is likely a significant source of HBsAg and HBx in these patients (38). In addition, it has important implications for HBV pathogenesis because HBx has been implicated in both the development and progression of HCC (39–41).

The N-terminal 20 residues of HBx (HBx_1–20_) are highly conserved in HBV as well as in HBx proteins from various mammalian hepadnaviruses (20). The N-terminal 58 residues are not required for the transactivation function of HBx (13), and the present study demonstrates that the N-terminal 44 residues are not required for Smc6 degradation and *trans*-complementation of HBVΔX transcription. Although the high degree of sequence conservation in this region may be due to a requirement to maintain the viral RNase H sequence in an overlapping reading frame (42), our data raise the question as to why the HBx open reading frame includes this apparently nonessential region. A potential explanation is that this region has a regulatory role, with the N-terminal 20 HBx residues being reported to *trans*-repress HBx-mediated transactivation (43, 44). Although our studies with lentivirus-expressed HBx do not demonstrate that N-terminally truncated HBx (HBx_31–154_ and HBx_45–154_) is more active than full-length HBx (HBx_1–154_), such a phenotype may be apparent only in the context of HBx expressed from cccDNA. Similarly, natural infection studies with the appropriate HBx mutants may be required to determine if deletion of the highly conserved N-terminal region modulates HBx expression or stability. Additional studies are therefore required to determine whether the HBx N terminus has a regulatory role or whether this highly conserved sequence has some alternate function.

Analysis of liver samples from HBV-positive HCC patients suggests that an alternative transcription start site (TSS) located within the HBx open reading frame between the first (M1) and second (M79) start codons may give rise to a shorter HBx protein (HBx_79–154_) (45). This shorter HBx protein has been reported to retain transactivation function (46). However, the observation from the present study that HBx_79–154_ is inactive suggests that translation initiation from M79 is not likely to generate a functional protein. Furthermore, mutation of M79 to alanine also did not influence HBx function as measured by Smc6 degradation and HBV transcription (data not shown). Similarly, the X protein from tent-making bat HBV has a leucine at this position but can still functionally substitute for HBx (20). Finally, cccDNA of HBVΔX is transcriptionally silent in PHH; this virus has a stop codon at residue 7 of HBx, and while it cannot produce full-length HBx, it can presumably produce HBx_79–154_ (4, 19). Collectively, these data suggest that this shorter HBx protein is not functional in the context of natural infection *in vitro*. However, because there is heterogeneity in TSS usage between patient liver samples and HBV-infected PHH (45), *in vivo* studies will likely be required to determine whether the shorter HBx protein plays a critical role in the HBV replication cycle.

In summary, the results reported here provide important new insights into the HBx structure-function relationship and suggest a new approach for HBx structural studies. Future studies will focus on determining the structure of this key viral regulatory protein.

## MATERIALS AND METHODS

### Expression and purification of DDB1.

The DDB1 construct (GenBank accession no. Q16531) was engineered to contain TEV (tobacco etch virus) protease-cleavable N-terminal His and GST tags (His-GST-TEV-DDB1) and cloned into the pFastBac1 vector. The recombinant protein was expressed from baculovirus-infected sf9 cells at a density of 1.5 × 10^6^ cells/ml in ESF-921 medium (Expression Systems, Davis, CA) and harvested at 70 h postinfection. A 1-liter cell pellet was suspended in 100 ml buffer A [25 mM Tris (pH 7.5), 300 mM NaCl, 0.5% (vol/vol) Triton X-100 (TX-100), and 2.5 mM Tris(2-carboxyethyl)phosphine hydrochloride (TCEP)] supplemented with two tablets of EDTA-free Complete protease inhibitor cocktail (Roche Diagnostics, Risch-Rotkreuz, Switzerland). Cells were lysed with a Dounce homogenizer and subjected to ultracentrifugation at 185,000 × *g*. The clarified supernatant was purified through glutathione resin equilibrated with buffer B (25 mM Tris [pH 7.5], 300 mM NaCl, 2.5 mM TCEP) and eluted with buffer C (25 mM Tris [pH 7.5], 300 mM NaCl, 2.5 mM TCEP, 20 mM reduced glutathione). Recombinant TEV protease was added to purified His-GST-TEV-DDB1, and the reaction was performed at 4°C overnight. The reaction mixture was applied to Ni affinity chromatography columns to remove the uncleaved protein, and the tag-removed fractions were pooled and further purified by size exclusion chromatography using a 120-ml Superdex 200 column (GE Healthcare, Little Chalfont, UK) equilibrated with buffer B. The molar mass and purity of DDB1 were confirmed by mass spectrometry.

### Expression and purification of the HBx-DDB1 fusion protein.

The HBx-DDB1 fusion protein is similar to the construct that was used to identify Smc5/6 as an HBx interaction partner (7). The HBx-DDB1 fusion construct (His-TEV-HBx-DDB1) encodes (from the amino terminus) a 10-His tag, a TEV protease cleavage site, HBx, a 12-residue linker (SGSGSGSGSGSG), and DDB1. His-TEV-HBx-DDB1 was cloned into the pFastBac1 vector. The HBx sequence of the HBx-DDB1 fusion protein and all other recombinant HBx proteins was the same as the sequence of HBx expressed in HepAD38 cells (genotype D; GenBank accession no. AUG90794). The fusion protein was expressed in sf9 cells at a density of 1.5 × 10^6^ cells/ml in ESF-921 medium, and cells were harvested at 70 h postinfection. A 1-liter cell pellet was suspended in 100 ml buffer A containing protease inhibitors, lysed with a Dounce homogenizer, and subjected to ultracentrifugation at 185,000 × *g*. The supernatant containing the solubilized His-TEV-HBx-DDB1 fusion protein was applied to a 5 ml Ni-nitrilotriacetic acid (NTA) column (GE Healthcare) equilibrated in buffer B. The column was washed with 50 ml of buffer B containing 20 mM imidazole, and His-TEV-HBx-DDB1 was eluted with a gradient of buffer B containing 500 mM imidazole over 50 ml. Fractions containing His-TEV-HBx-DDB1 were pooled, buffer exchanged to buffer D (25 mM Tris [pH 7.5], 50 mM NaCl, 2.5 mM TCEP), and applied to an 8-ml MonoQ column (GE Healthcare). His-TEV-HBx-DDB1 was eluted using a linear gradient from 50 mM to 1 M NaCl. Fractions containing His-TEV-HBx-DDB1 were pooled, and TEV protease was added and incubated overnight at 4°C, followed by the use of a Ni-NTA column to remove the uncleaved protein. The flowthrough from the Ni-NTA column was concentrated and further purified by size exclusion chromatography using a 24-ml Superdex 200 column (GE Healthcare) equilibrated in buffer B. The molar mass and purity of the HBx-DDB1 fusion protein were confirmed by mass spectrometry analysis.

### Expression and purification of the HBx:DDB1 coexpressed complex.

Wild-type HBx (genotype D) was cloned into the pFastbac1 vector. The baculoviruses of HBx and His-GST-TEV-DDB1 were used to coinfect sf9 cells. The cells were harvested at 64 h postinfection and lysed in buffer A containing protease inhibitors as described above. The clarified supernatant was applied to glutathione resin equilibrated with buffer B and eluted with buffer C. The purified HBx:His-GST-TEV-DDB1 complex was subjected to TEV protease cleavage at 4°C overnight and applied to a Ni affinity chromatography column to remove the uncleaved protein complex. The untagged fractions were pooled, equilibrated in buffer D, and applied to an 8-ml MonoQ column. The HBx:DDB1 complex was eluted using a linear gradient from 50 mM to 1 M NaCl. Fractions containing both HBx and DDB1 were pooled and further purified by size exclusion chromatography using a 24-ml Superdex 200 column (GE Healthcare) equilibrated in buffer B. The molar mass and purity of the HBx:DDB1 complex were confirmed by mass spectrometry.

### Expression and purification of the His-HBx:GST-DDB1 coexpressed complex.

Wild-type HBx (genotype D) was cloned into the pFastBacHTA vector. DDB1 was engineered with a GST tag at the N terminus and cloned into the pFastBac1 vector. The baculoviruses of His-HBx and GST-DDB1 were used to coinfect sf9 cells. The cells were harvested at 64 h postinfection and lysed in buffer A containing protease inhibitors as described above. The clarified supernatant was applied to glutathione resin equilibrated with buffer B and eluted with buffer C. The purified His-HBx:GST-DDB1 complex was applied to a 5-ml Ni-NTA column (GE Healthcare) equilibrated in buffer B. The column was washed with 50 ml of buffer B containing 20 mM imidazole and eluted with a gradient of buffer B containing 500 mM imidazole over 50 ml for further purification. Fractions containing both His-HBx and GST-DDB1 were pooled and further purified by size exclusion chromatography using a 24-ml Superdex 200 column (GE Healthcare) equilibrated in buffer B. The molar mass and purity of the His-HBx:GST-DDB1 complex were confirmed by mass spectrometry.

### Expression and purification of His-HBx and SV5-V.

His-HBx, encoding wild-type HBx (genotype D) preceded by a His tag and a TEV cleavage site, was generated by PCR. The PCR fragment was ligated into pET-28a. His-HBx was expressed in E. coli Rosetta2(DE3) cells. Cells were grown in LB at 37°C, and expression was induced with 1 mM isopropyl-β-d-thiogalactopyranoside (IPTG) for 12 h at 16°C. Cells were lysed in phosphate-buffered saline (PBS) supplemented with 1 mM dithiothreitol (DTT). The membrane fraction was isolated by centrifugation and solubilized with 1% Fos-choline 14 (FC14). The solubilized membranes were loaded onto a HiTrap SP column, and the column was washed with a solution containing 20 mM morpholineethanesulfonic acid (MES) (pH 6), 1 mM DTT, and 0.01% FC14 containing 100, 200, and 300 mM NaCl. His-HBx was eluted with 1 M NaCl. His-HBx-containing fractions were pooled, concentrated, and applied to a 24-ml Superdex 200 column equilibrated in a solution containing 20 mM HEPES (pH 7.2), 150 mM NaCl, 0.5 mM TCEP, and 0.01% FC14. The molar mass and purity of HIS-HBx were confirmed by mass spectrometry.

pGEX-SV5-V, encoding SV5-V (GenBank accession no. P11207) preceded by GST and a thrombin cleavage site, was generated by PCR. The PCR fragment was ligated into pGEX-4T1. GST–SV5-V was expressed in Rosetta cells. E. coli cells were grown in LB at 37°C, and expression was induced with 0.5 mM IPTG for 24 h at 16°C. E. coli cells were lysed in a solution containing 25 mM Tris (pH 8.5), 150 mM NaCl, 0.01% NaN_3_, and 1 mM DTT, supplemented with 1% TX-100. The soluble fraction was loaded onto glutathione agarose and eluted with 20 mM reduced glutathione. GST–SV5-V was incubated with thrombin and reapplied to glutathione agarose to separate SV5-V from GST and uncleaved GST–SV5-V. SV5-V was loaded onto a MonoQ column and eluted with a linear gradient from 50 to 1,000 mM NaCl. Fractions containing SV5-V were pooled and applied to a 24-ml Superdex 75 column equilibrated in a solution containing 25 mM Tris (pH 8.5), 150 mM NaCl, 0.01% NaN_3_, and 1 mM DTT. The molar mass and purity of SV5-V were confirmed by mass spectrometry.

### Analytical ultracentrifugation.

Sedimentation velocity experiments with His-HBx, DDB1, the HBx-DDB1 fusion, and the HBx:DDB1 coexpressed complex were performed at 20°C in a ProteomeLab XL-A analytical ultracentrifuge (Beckman Coulter, Inc., Fullerton, CA). Samples of 400 μl in buffer identical to the final storage buffer for each protein with the exception of 0.01% NaN_3_ were loaded into a dual-sector charcoal-filled Epon centerpiece. Samples were centrifuged at 128,794 × *g* or 141,995 × *g* with an An50-Ti rotor, and sedimentation was monitored by the absorbance at a wavelength of 280 nm for each specific protein. Data were analyzed with the SEDFIT program (NIH, Bethesda, MD), which generated a continuous *c*(*s*) distribution for the sedimenting species for each protein analyzed.

### Dynamic light scattering.

The percent polydispersity (%Pd) values of SV5-V, DDB1, the HBx-DDB1 fusion, and the HBx:DDB1 complex were analyzed by dynamic light scattering (DLS) using a DynaPro Titan system (Wyatt Technology Corporation, Santa Barbara, CA). Each protein was diluted to 1 mg/ml and centrifuged at 18,800 × *g* for 10 min at 4°C prior to DLS measurements. Each sample was equilibrated at room temperature for 5 min and measured in triplicate, in which a single measurement consists of 10 acquisitions for 10 s at ambient temperature using DYNAMICS v6.12.0.3 software. The raw data were analyzed by distribution plots, and the %Pd was obtained from regularization analysis of each protein sample.

### Time-resolved fluorescence resonance energy transfer.

The interaction between His-HBx and GST-DDB1 (expressed individually or coexpressed) was measured in the absence and presence of competing untagged proteins (HBx, DDB1, or SV5-V) or peptides (HBx H-box wild-type or R96E mutant peptide) by time-resolved fluorescence resonance energy transfer (TR-FRET). Assays evaluating the interaction of individually expressed His-HBx and GST-DDB1 were performed with a 75-μl final volume in a white 96-well microplate (Corning, Corning, NY) in buffer containing 25 mM HEPES (pH 7.2), 400 mM NaCl, 0.1 mg/ml BSA (Roche Diagnostics), 0.01% (vol/vol) Brij-35 (MilliporeSigma, St. Louis, MO), and 0.5 mM TCEP (MilliporeSigma). Briefly, 5 nM His-HBx and 5 nM GST-DDB1 were preincubated in the absence and presence of increasing concentrations of untagged proteins or peptides for 30 min at room temperature. Complex formation between His-HBx and GST-DDB1 was detected by the addition of fluorescently labeled detection antibodies following a 1-h incubation at room temperature. Eu-cryptate-conjugated anti-His antibody (fluorescence donor; Cisbio, Bedford, MA) and d2-conjugated anti-GST antibody (fluorescence acceptor; Cisbio) were used at concentrations of 1.78 nM and 6.67 nM, respectively. TR-FRET was measured on Tecan M1000 plate reader (excitation at 320 nm and emission at 615 nm and 665 nm).

Assays evaluating the interaction of the His-HBx:GST-DDB1 complex were performed with a 10-μl final volume in a white 384-well microplate (Corning) in buffer containing 20 mM HEPES (pH 7.2), 50 mM NaCl, 0.1 mg/ml BSA (Roche Diagnostics), 0.01% (vol/vol) Brij-35 (MilliporeSigma), and 0.5 mM TCEP (MilliporeSigma). Briefly, 3 nM the His-HBx:GST-DDB1 complex was preincubated in the absence and presence of increasing concentrations of untagged proteins or peptides for 1 h at room temperature. Complex formation between His-HBx and GST-DDB1 was detected by the addition of fluorescently labeled detection antibodies following a 1-h incubation at room temperature. Eu-cryptate-conjugated anti-His antibody (fluorescence donor; Cisbio, Bedford, MA) and d2-conjugated anti-GST antibody (fluorescence acceptor; Cisbio) were used at concentrations of 1 nM and 2.5 nM, respectively. TR-FRET was measured on an EnVision plate reader (excitation at 320 nm and emission at 620 nm and 665 nm).

For both assay formats, binding of His-HBx to GST-DDB1 results in an increase of TR-FRET between a donor and an acceptor, whereas the displacement of one of the interacting proteins by an untagged competitor protein or a peptide results in a decrease in TR-FRET. The dose response (ratio of the fluorescence intensities at 665 nm and 615/620 nm as a function of the competitor concentration) was analyzed using a 4-parameter logistic equation to calculate IC_50_ values.

### Inductively coupled plasma mass spectrometry.

Inductively coupled plasma mass spectrometry (ICP-MS) was performed by Pacific Biolabs (Hercules, CA) using an Agilent Technologies 7700 series ICP-MS instrument (Agilent Technologies, Santa Clara, CA). For quantitative analysis, samples were diluted 1 in 10 with an internal standard solution. An eight-point calibration curve ranging from 1 ppb to 1,000 ppb was used to estimate the concentration of the analyte in the samples. All dilutions were made with pure Millipore water that had been generated with a MilliQ water purification system (∼18 MΩ cm). To minimize the memory effects of the analyte, a rinse solution comprised of 4% tetramethylammonium hydroxide (TMAH)–0.3% EDTA–0.4% Triton X-100 was injected after calibration standards and quality controls (QCs) and between the samples. Purified proteins were analyzed for the presence of iron and zinc. Protein concentrations were determined by the absorbance at 280 nm using an extinction coefficient calculated from the amino acid composition.

### Hydrogen-deuterium exchange mass spectrometry.

Continuous hydrogen-deuterium exchange (HDX) labeling of the HBx:DDB1 complex was performed by taking 70 pmol of the complex and exchanging it in D_2_O (95%) at pH 7.4 at 25°C in 25 mM Tris buffer for 0, 10, 30, 60, 300, 900, 3,600, 14,400, and 86,400 s, as previously described (47). The same protocol was used for the HBx-DDB1 fusion protein. To remove the native zinc, the complex was incubated with 5 mM EDTA for 2 h at 37°C prior to HDX. Quenching was performed under reducing conditions by adding a solution of 4 M urea with 1% trifluoroacetic acid (TFA) to the reaction vial at a 1:1.5 (vol/vol) protein/quench ratio. The final pH was kept at approximately 2.5 to minimize back-exchange. The samples were mixed and immediately loaded onto a custom-built HDX platform for desalting, online pepsin digestion, reversed-phase chromatography of peptic fragments, and direct injection into the mass spectrometer for analysis.

The samples were passed over a custom-packed 2-mm by 20-mm immobilized pepsin column at a 200-μl/min flow rate (48). The peptides resulting from digestion were captured by a 2.1-mm by 5-mm Opti-Lynx peptide microtrap column (Optimize Technologies, Oregon City, OR) and desalted at 200 μl/min with H_2_O containing 0.1% trifluoroacetic acid for 3 min. The resulting peptides were then separated by a 2.1-mm by 50-mm C_18_ column (2.5-μm XSelect charged surface hybrid [CSH] C_18_) with a 13-min gradient of 5 to 80% acetonitrile in 0.1% formic acid at a flow rate of 100 μl/min delivered by a high-performance liquid chromatography (HPLC) pump (Shimadzu Scientific Instruments, Kyoto, Japan). The linear part of the gradient from 1.5 to 8.5 min raised the acetonitrile content from 15% to 50%, during which time most of the peptides eluted from the C_18_ column. The entire fluidic system, except the pepsin column, was kept in an ice bath to minimize back-exchange. Duplicate measurements were carried out for each time point. MS detection was performed on a Thermo LTQ XL Orbitrap instrument (Thermo Fisher Scientific, Waltham, MA) using the following instrument parameters: spray voltage of 4.8 kV, capillary temperature of 280°C, capillary voltage of 41.5 V, and tube lens at 145 V. Data were collected at a mass resolving power of 100,000 at *m/z* 400.

The collated peptide list from multiple MS2 runs and the MS1 raw files from nondeuterated runs were analyzed using HDX Workbench (Omics Informatics, Honolulu, HI) (49). The centroid masses of isotopic envelopes and deuterium levels were determined as described previously (50, 51).

### NEM chemical footprinting.

For NEM labeling, the coexpressed HBx:DDB1 complex both with native zinc and after zinc removal (EDTA treatment) was incubated with 1 mM NEM (protein:NEM at a 1:10 molar ratio) in 1× PBS (pH 7.4) at room temperature. Time-dependent labeling was performed in triplicate, and the reactions were quenched by the addition of 20 mM DTT after 30, 60, 120, 300, and 600 s. The labeled samples were digested overnight with chymotrypsin (Promega, Madison, WI) at 37°C with a protease-to-protein ratio of 1:20 (wt/wt) by using a previously described protocol (52). The digestion was terminated by adding 1% TFA. Approximately 5 pmol of digested peptides was injected for liquid chromatography-tandem mass spectrometry (LC-MS/MS) analysis. Samples were first desalted on an Acclaim PepMap C_18_ column (105 μm by 2 cm, 5 μm, and 100 Å; Thermo Fisher Scientific) for 10 min. Separation was performed on a custom-packed UChrom C_18_ column (75 μm by 15 cm, 3 μm, and 200 Å; NanoLCMS Solutions, Oroville, CA) using an UltiMate 3000 RSLCnano system (Thermo Fisher Scientific). Peptides were eluted at a flow rate of 500 nl/min with the following gradient: 2% buffer B to 20% in 47 min, which was increased to 50% in 35 min and then to 90% in 15 min, held at 90% for 10 min, returned to 2% buffer B in 1 min, and equilibrated at 2% buffer B for 15 min. The flow was directed to a Nanospray Flex source coupled with a Q Exactive Plus Orbitrap mass spectrometer (Thermo Fisher Scientific) with a spray voltage of 3.2 kV and a capillary temperature of 200°C. In the data-dependent mode, the 10 most-abundant ions were selected for higher-energy collisional dissociation (HCD) with an isolation window of *m/z* 1.5 and a normalized collision energy of 30%.

NEM modification of cysteine-containing peptides was determined with Byonic (Protein Metrics, Cupertino, CA). Modification fractions for cysteine-containing peptides were interrogated with Byologic (Protein Metrics). Modification sites on the peptide were assigned on the basis of product ion spectra (MS/MS data) and were manually verified. Extracted ion chromatograms (XICs) were used to determine ratios of NEM modification for a particular cysteine residue by dividing the intensities of the modified peptides/residue (*I*_NEM_) by the summed intensity of modified and unmodified peptides (*I*) [i.e., % modification = Σ*I*_NEM_/(Σ*I*_NEM_ + Σ*I*)].

### Liquid chromatography-tandem mass spectrometry analysis.

Huh-7 or HEK293T cells grown to 90% confluence in six 100-mm plates were transiently transfected with the pcDNA4-HA-HBx plasmid using GenJet (SignaGen Laboratories, Rockville, MD) or X-tremeGENE HP DNA transfection reagent (Roche Diagnostics), respectively, according to the manufacturer’s instructions. HA-tagged HBx was expressed for 36 h, and cells were harvested by lysis in buffer containing 0.15 M NaCl, 50 mM Tris (pH 7.5), 1% Triton X-100, and 1% deoxycholate (IP buffer). IP buffer (2 ml) was added to each 100-mm plate to lyse cells for 30 min at 4°C. Cellular debris and nuclei were removed by centrifugation for 10 min at 14,000 × *g* at 4°C. Precleared lysates were pooled, and HA-HBx protein was immunoprecipitated using 150 μl of Pierce anti-HA magnetic beads (Thermo Fisher Scientific) overnight at 4°C. Immunoprecipitates were washed three times with IP buffer and three times with IP buffer containing no detergents. Proteins were then either reduced by dithiothreitol and alkylated by iodoacetamide or left in their native form and then digested with trypsin overnight at pH 8.5. The resulting peptides were analyzed with an UltiMate 3000 RSLCnano system (Thermo Fisher Scientific) coupled to a TripleTOF 5600 mass spectrometer with a NanoSpray III source (Sciex, Redwood City, CA). The peptides were trapped and desalted with 2% acetonitrile in 0.1% formic acid at flow rate of 5 μl/min on an Acclaim PepMap100 column (5 μm, 2-cm by 100-μm internal diameter [ID]; Thermo Fisher Scientific). Eluted peptides were separated using an Acclaim PepMap100 analytical column (3 μm, 25-cm by 75-μm ID; Thermo Fisher Scientific). The 70 -min elution gradient at a constant flow rate of 300 nl/min was set to 5% phase B (0.1% formic acid in 99.9% acetonitrile) (phase A, 0.1% formic acid) for the first 10 min and then gradient elution by increasing the content of acetonitrile. The time of flight (TOF) mass spectrometer mass range was set to *m/z* 350 to 1,250, in the MS/MS mode, and the instrument acquired fragmentation spectra for ions of *m/z* 100 to 1,600. Protein Pilot 4.5 (Sciex) was used for peptide and protein identification using a database of sequences of HA-tagged HBx, Swiss-Prot human proteins (downloaded on 15 February 2016), and common contaminants.

### HBV virion production and PHH infection.

Production of HBVΔX virions from the HepG2-H1.3×^−^ stable cell line was performed as previously described (19). Lentiviruses stably expressing the c-Myc-tagged HBx-DDB1 fusion, wild-type HBx, or mutated or truncated versions of HBx (genotype D) were prepared using cDNA expression vectors with a cytomegalovirus (CMV) promoter (System Biosciences, Palo Alto, CA). The sequence of the HBx-DDB1 fusion was identical to that of the recombinant HBx-DDB1 protein. Cryopreserved PHH isolated from livers of deceased donors were purchased from Life Technologies (Grand Island, NY). Consent was obtained from the donor or the donor’s legal next of kin for use of these samples and their derivatives for research purposes using institutional review board (IRB)-approved authorizations. PHH were thawed and then seeded in cell plating medium (Life Technologies) at a density of 65,000 cells per well in 96-well collagen-coated plates (Life Technologies). At 24 h postplating, the plating medium was replaced with maintenance medium (Life Technologies) containing 1.5% dimethyl sulfoxide (DMSO) and 2% fetal bovine serum (FBS), and the cells were then incubated at 37°C in a humidified 5% CO_2_ incubator. PHH were incubated in maintenance medium for 1 day before lentiviral transduction (multiplicity of infection [MOI] of 3) in maintenance medium containing 8 μg/ml Polybrene. The cells were then evaluated by Western blotting or were infected with HBVΔX. For Western blot studies, at 72 h posttransduction, the cell lysates were generated by adding 250 μl 1× cell lysis buffer (Cell Signaling, Danvers, MA) supplemented with a protease inhibitor cocktail (Thermo Fisher Scientific) and then scraping the wells using a cell scraper. The lysates were sonicated for 30 s, centrifuged for 1 min at 21,130 × *g*, and then analyzed by Western blotting. For the infection studies, 1 day after lentivirus transduction, medium was removed, and cells were infected with HBVΔX virions (genotype D) at 500 to 1,000 viral genome equivalents per cell in medium containing 4% polyethylene glycol 8000 (PEG 8000) for 16 h at 37°C. Mock-infected cells were treated with medium containing 4% PEG 8000 in the absence of virus. At the end of the infection period, cells were washed three times with Williams’ E medium (Thermo Fisher Scientific) and cultured in maintenance medium containing 1.5% DMSO and 2% FBS. The PHH medium was subsequently changed every 3 to 4 days until the end of the study.

### Western blotting.

Western blot analysis of PHH was performed as previously described (19). Membranes were probed with a 1:250 dilution of monoclonal mouse anti-human Smc6 (clone 2E7/M01) (Abgent, San Diego, CA), a 1:1,000 dilution of mouse monoclonal anti-Myc (clone 9E10) (LifeSpan Biosciences, Seattle, WA), and a 1:2,000 dilution of rabbit monoclonal anti-glyceraldehyde-3-phosphate dehydrogenase (GAPDH) (clone 14C10) (Cell Signaling). IRDye 680RD goat anti-rabbit or IRDye 800CW goat anti-mouse IgG (Li-Cor, Lincoln, NE) at a 1:5,000 dilution was used as a secondary antibody. Blots were visualized using an Odyssey infrared imaging system (Li-Cor).

### Coimmunoprecipitation assay.

PHH were plated onto 6-well plates at a density of 1.5 million cells per well. The cells were transduced with GFP- or HBx-expressing lentiviruses (MOI of 3) 24 h after plating. At 72 h posttransduction, cell lysates were generated by adding 250 μl 1× cell lysis buffer (Cell Signaling) supplemented with a protease inhibitor cocktail (Thermo Fisher Scientific) and then scraping the wells using a cell scraper. The lysates were sonicated for 30 s and centrifuged for 1 min at 21,130 × *g*. The supernatants were collected, and 65 μl of each was set aside for Western blotting. Anti-DDB1 antibody (clone 5428) (Cell Signaling) was added to the remainder of the supernatant at a 1:250 dilution, and the samples were mixed gently with rocking overnight at 4°C. Protein A/G magnetic beads (Thermo Fisher Scientific) were washed according to the manufacturer’s protocol, added to the lysate, and then incubated for 1 h at room temperature. The magnetic beads were washed three times using 1× cell lysis buffer (Cell Signaling) and then once with deionized water. Proteins bound to the beads were eluted by boiling in SDS-PAGE buffer for 10 min. The eluted proteins were subsequently electrophoresed for 1.5 h at 150 V on a NuPage 4-to-12% gradient gel in 1× MES buffer (Thermo Fisher Scientific) and Western blotted as described above. HBx was detected using an anti-Myc antibody.

### HBeAg analysis.

Hepatitis B virus e antigen (HBeAg) was detected in culture media by an electrochemiluminescence assay (MSD). The HBeAg concentration in each sample was calculated by interpolation from a standard curve with purified HBeAg. The MSD assay was performed according to the manufacturer’s instructions (Meso Scale Diagnostics, Rockville, MD). Briefly, cultured supernatants were inactivated with 0.5% Triton X-100 (30 min at 37°C) and then transferred into plates prespotted with an anti-HBeAg capture antibody (clone 773; GenWay Bio, San Diego, CA). The plates were then incubated overnight at 4°C. On the next day, the plates were washed three times with 1× PBS with 0.5% Tween, and an anti-HBeAg detection antibody (clone 774; GenWay Bio) was added to each well. Following a 2-h incubation at room temperature with fast shaking, the plates were washed with 1× PBS with 0.5% Tween, and a 2× solution of MSD read buffer T was added. The plates were then read on a Sector Imager 6000 plate scanner.

### HBV sequence analysis.

A set of 7,253 HBx sequences (genotypes A to H) was downloaded from the March 2017 version of the HBV database (53), aligned using BioEdit (54), and analyzed using Pipeline Pilot (Biovia, San Diego, CA). The HBx sequence (GenBank accession no. AUG90794; genotype D) was also compared to the X protein sequences from woodchuck hepatitis virus (GenBank accession no. P03167) as well as the X coding sequences from hepadnaviruses infecting the New World woolly monkey (GenBank accession no. O71302), the roundleaf bat (GenBank accession no. U3M9Y2), the horseshoe bat (GenBank accession no. U3M9X8), and the tent-making bat (GenBank accession no. YP_009046001.1).

### Statistical analysis.

Data are expressed as means ± standard errors of the means (SEM) unless otherwise stated. Statistical significance was tested using a two-tailed *t* test (for two-sample comparisons) or one-way analysis of variance (ANOVA) with multiple-comparison correction (for multiple comparisons). A *P* value of <0.05 was considered significant.

## Supplementary Material

Supplemental file 1

## References

[1] Schweitzer A, Horn J, Mikolajczyk RT, Krause G, Ott JJ 2015 Estimations of worldwide prevalence of chronic hepatitis B virus infection: a systematic review of data published between 1965 and 2013. Lancet 386:1546–1555. doi:10.1016/S0140-6736(15)61412-X. 26231459

[2] Lozano R, Naghavi M, Foreman K, Lim S, Shibuya K, Aboyans V, Abraham J, Adair T, Aggarwal R, Ahn SY, Alvarado M, Anderson HR, Anderson LM, Andrews KG, Atkinson C, Baddour LM, Barker-Collo S, Bartels DH, Bell ML, Benjamin EJ, Bennett D, Bhalla K, Bikbov B, Bin Abdulhak A, Birbeck G, Blyth F, Bolliger I, Boufous S, Bucello C, Burch M, Burney P, Carapetis J, Chen H, Chou D, Chugh SS, Coffeng LE, Colan SD, Colquhoun S, Colson KE, Condon J, Connor MD, Cooper LT, Corriere M, Cortinovis M, de Vaccaro KC, Couser W, Cowie BC, Criqui MH, Cross M, Dabhadkar KC, 2012 Global and regional mortality from 235 causes of death for 20 age groups in 1990 and 2010: a systematic analysis for the Global Burden of Disease Study 2010. Lancet 380:2095–2128. doi:10.1016/S0140-6736(12)61728-0. 23245604 PMC10790329

[3] Kornyeyev D, Voitenleitner C, Livingston CM, Xing W, Hung M, Kwon HJ, Fletcher SP, Beran RK 2019 Spatiotemporal analysis of hepatitis B virus X protein in primary human hepatocytes. J Virol 93:e00248-19. doi:10.1128/JVI.00248-19. 31167911 PMC6675897

[4] Lucifora J, Arzberger S, Durantel D, Belloni L, Strubin M, Levrero M, Zoulim F, Hantz O, Protzer U 2011 Hepatitis B virus X protein is essential to initiate and maintain virus replication after infection. J Hepatol 55:996–1003. doi:10.1016/j.jhep.2011.02.015. 21376091

[5] Riviere L, Gerossier L, Ducroux A, Dion S, Deng Q, Michel ML, Buendia MA, Hantz O, Neuveut C 2015 HBx relieves chromatin-mediated transcriptional repression of hepatitis B viral cccDNA involving SETDB1 histone methyltransferase. J Hepatol 63:1093–1102. doi:10.1016/j.jhep.2015.06.023. 26143443

[6] Belloni L, Pollicino T, De Nicola F, Guerrieri F, Raffa G, Fanciulli M, Raimondo G, Levrero M 2009 Nuclear HBx binds the HBV minichromosome and modifies the epigenetic regulation of cccDNA function. Proc Natl Acad Sci U S A 106:19975–19979. doi:10.1073/pnas.0908365106. 19906987 PMC2775998

[7] Decorsière A, Mueller H, van Breugel PC, Abdul F, Gerossier L, Beran RK, Livingston CM, Niu C, Fletcher SP, Hantz O, Strubin M 2016 Hepatitis B virus X protein identifies the Smc5/6 complex as a host restriction factor. Nature 531:386–389. doi:10.1038/nature17170. 26983541

[8] Murphy CM, Xu Y, Li F, Nio K, Reszka-Blanco N, Li X, Wu Y, Yu Y, Xiong Y, Su L 2016 Hepatitis B virus X protein promotes degradation of SMC5/6 to enhance HBV replication. Cell Rep 16:2846–2854. doi:10.1016/j.celrep.2016.08.026. 27626656 PMC5078993

[9] van Breugel PC, Robert EI, Mueller H, Decorsiere A, Zoulim F, Hantz O, Strubin M 2012 Hepatitis B virus X protein stimulates gene expression selectively from extrachromosomal DNA templates. Hepatology 56:2116–2124. doi:10.1002/hep.25928. 22744635

[10] Leupin O, Bontron S, Schaeffer C, Strubin M 2005 Hepatitis B virus X protein stimulates viral genome replication via a DDB1-dependent pathway distinct from that leading to cell death. J Virol 79:4238–4245. doi:10.1128/JVI.79.7.4238-4245.2005. 15767425 PMC1061538

[11] Li T, Robert EI, van Breugel PC, Strubin M, Zheng N 2010 A promiscuous alpha-helical motif anchors viral hijackers and substrate receptors to the CUL4-DDB1 ubiquitin ligase machinery. Nat Struct Mol Biol 17:105–111. doi:10.1038/nsmb.1719. 19966799 PMC2823288

[12] Lin-Marq N, Bontron S, Leupin O, Strubin M 2001 Hepatitis B virus X protein interferes with cell viability through interaction with the p127-kDa UV-damaged DNA-binding protein. Virology 287:266–274. doi:10.1006/viro.2001.1036. 11531405

[13] Kumar V, Jayasuryan N, Kumar R 1996 A truncated mutant (residues 58-140) of the hepatitis B virus X protein retains transactivation function. Proc Natl Acad Sci U S A 93:5647–5652. doi:10.1073/pnas.93.11.5647. 8643631 PMC39302

[14] Hodgson AJ, Hyser JM, Keasler VV, Cang Y, Slagle BL 2012 Hepatitis B virus regulatory HBx protein binding to DDB1 is required but is not sufficient for maximal HBV replication. Virology 426:73–82. doi:10.1016/j.virol.2012.01.021. 22342275 PMC3294142

[15] Jiang T, Liu M, Wu J, Shi Y 2016 Structural and biochemical analysis of Bcl-2 interaction with the hepatitis B virus protein HBx. Proc Natl Acad Sci U S A 113:2074–2079. doi:10.1073/pnas.1525616113. 26858413 PMC4776483

[16] Fischer ES, Bohm K, Lydeard JR, Yang H, Stadler MB, Cavadini S, Nagel J, Serluca F, Acker V, Lingaraju GM, Tichkule RB, Schebesta M, Forrester WC, Schirle M, Hassiepen U, Ottl J, Hild M, Beckwith RE, Harper JW, Jenkins JL, Thoma NH 2014 Structure of the DDB1-CRBN E3 ubiquitin ligase in complex with thalidomide. Nature 512:49–53. doi:10.1038/nature13527. 25043012 PMC4423819

[17] Li T, Chen X, Garbutt KC, Zhou P, Zheng N 2006 Structure of DDB1 in complex with a paramyxovirus V protein: viral hijack of a propeller cluster in ubiquitin ligase. Cell 124:105–117. doi:10.1016/j.cell.2005.10.033. 16413485

[18] Schwefel D, Groom HC, Boucherit VC, Christodoulou E, Walker PA, Stoye JP, Bishop KN, Taylor IA 2014 Structural basis of lentiviral subversion of a cellular protein degradation pathway. Nature 505:234–238. doi:10.1038/nature12815. 24336198 PMC3886899

[19] Niu C, Livingston CM, Li L, Beran RK, Daffis S, Ramakrishnan D, Burdette D, Peiser L, Salas E, Ramos H, Yu M, Cheng G, Strubin M, Delaney WE IV, Fletcher SP 2017 The Smc5/6 complex restricts HBV when localized to ND10 without inducing an innate immune response and is counteracted by the HBV X protein shortly after infection. PLoS One 12:e0169648. doi:10.1371/journal.pone.0169648. 28095508 PMC5240991

[20] Abdul F, Filleton F, Gerossier L, Paturel A, Hall J, Strubin M, Etienne L 2018 Smc5/6 antagonism by HBx is an evolutionarily conserved function of hepatitis B virus infection in mammals. J Virol 92:e00769-18. doi:10.1128/JVI.00769-18. 29848586 PMC6069175

[21] Urban S, Hildt E, Eckerskorn C, Sirma H, Kekule A, Hofschneider PH 1997 Isolation and molecular characterization of hepatitis B virus X-protein from a baculovirus expression system. Hepatology 26:1045–1053. doi:10.1002/hep.510260437. 9328333

[22] Sidhu K, Kumar S, Reddy VS, Kumar V 2014 Mass spectrometric determination of disulfide bonds in the biologically active recombinant HBx protein of hepatitis B virus. Biochemistry 53:4685–4695. doi:10.1021/bi500140t. 24971648

[23] Feige MJ, Hendershot LM 2011 Disulfide bonds in ER protein folding and homeostasis. Curr Opin Cell Biol 23:167–175. doi:10.1016/j.ceb.2010.10.012. 21144725 PMC3078216

[24] Nakatani T, Tawaramoto M, Opare Kennedy D, Kojima A, Matsui-Yuasa I 2000 Apoptosis induced by chelation of intracellular zinc is associated with depletion of cellular reduced glutathione level in rat hepatocytes. Chem Biol Interact 125:151–163. doi:10.1016/S0009-2797(99)00166-0. 10731516

[25] Hashemi M, Ghavami S, Eshraghi M, Booy EP, Los M 2007 Cytotoxic effects of intra and extracellular zinc chelation on human breast cancer cells. Eur J Pharmacol 557:9–19. doi:10.1016/j.ejphar.2006.11.010. 17169355

[26] Grant K, Grant L, Tong L, Boutell C 2012 Depletion of intracellular zinc inhibits the ubiquitin ligase activity of viral regulatory protein ICP0 and restricts herpes simplex virus 1 replication in cell culture. J Virol 86:4029–4033. doi:10.1128/JVI.06962-11. 22278229 PMC3302540

[27] Guo Y, Dong L, Qiu X, Wang Y, Zhang B, Liu H, Yu Y, Zang Y, Yang M, Huang Z 2014 Structural basis for hijacking CBF-beta and CUL5 E3 ligase complex by HIV-1 Vif. Nature 505:229–233. doi:10.1038/nature12884. 24402281

[28] Becker SA, Lee TH, Butel JS, Slagle BL 1998 Hepatitis B virus X protein interferes with cellular DNA repair. J Virol 72:266–272. 9420223 10.1128/jvi.72.1.266-272.1998 PMC109372

[29] Lee SH, Cha EJ, Lim JE, Kwon SH, Kim DH, Cho H, Han KH 2012 Structural characterization of an intrinsically unfolded mini-HBX protein from hepatitis B virus. Mol Cells 34:165–169. doi:10.1007/s10059-012-0060-z. 22820921 PMC3887815

[30] Tu T, Budzinska MA, Shackel NA, Urban S 2017 HBV DNA integration: molecular mechanisms and clinical implications. Viruses 9:E75. doi:10.3390/v9040075. 28394272 PMC5408681

[31] Liu XH, Lin J, Zhang SH, Zhang SM, Feitelson MA, Gao HJ, Zhu MH 2008 COOH-terminal deletion of HBx gene is a frequent event in HBV-associated hepatocellular carcinoma. World J Gastroenterol 14:1346–1352. doi:10.3748/wjg.14.1346. 18322946 PMC2693680

[32] Tu H, Bonura C, Giannini C, Mouly H, Soussan P, Kew M, Paterlini-Bréchot P, Bréchot C, Kremsdorf D 2001 Biological impact of natural COOH-terminal deletions of hepatitis B virus X protein in hepatocellular carcinoma tissues. Cancer Res 61:7803–7810. 11691796

[33] Ma NF, Lau SH, Hu L, Xie D, Wu J, Yang J, Wang Y, Wu MC, Fung J, Bai X, Tzang CH, Fu L, Yang M, Su YA, Guan XY 2008 COOH-terminal truncated HBV X protein plays key role in hepatocarcinogenesis. Clin Cancer Res 14:5061–5068. doi:10.1158/1078-0432.CCR-07-5082. 18698024

[34] Arii M, Takada S, Koike K 1992 Identification of three essential regions of hepatitis B virus X protein for trans-activation function. Oncogene 7:397–403. 1549357

[35] Renner M, Haniel A, Burgelt E, Hofschneider PH, Koch W 1995 Transactivating function and expression of the x gene of hepatitis B virus. J Hepatol 23:53–65. doi:10.1016/0168-8278(95)80311-4. 8530810

[36] Kim YH, Kang SK, Lee YI 1993 Functional analysis of hepatitis B virus transactivator X: implication of the leucine zipper-like region and C-terminal seven conserved amino acids in functional regions. Biochem Biophys Res Commun 197:894–903. doi:10.1006/bbrc.1993.2563. 8267629

[37] Takada S, Koike K 1990 Trans-activation function of a 3′ truncated X gene-cell fusion product from integrated hepatitis B virus DNA in chronic hepatitis tissues. Proc Natl Acad Sci U S A 87:5628–5632. doi:10.1073/pnas.87.15.5628. 2165598 PMC54380

[38] Wooddell CI, Yuen MF, Chan HL, Gish RG, Locarnini SA, Chavez D, Ferrari C, Given BD, Hamilton J, Kanner SB, Lai CL, Lau JYN, Schluep T, Xu Z, Lanford RE, Lewis DL 2017 RNAi-based treatment of chronically infected patients and chimpanzees reveals that integrated hepatitis B virus DNA is a source of HBsAg. Sci Transl Med 9:eaan0241. doi:10.1126/scitranslmed.aan0241. 28954926 PMC5830187

[39] Levrero M, Zucman-Rossi J 2016 Mechanisms of HBV-induced hepatocellular carcinoma. J Hepatol 64:S84–S101. doi:10.1016/j.jhep.2016.02.021. 27084040

[40] Ringelhan M, Protzer U 2015 Oncogenic potential of hepatitis B virus encoded proteins. Curr Opin Virol 14:109–115. doi:10.1016/j.coviro.2015.08.015. 26426688

[41] Livingston CM, Ramakrishnan D, Strubin M, Fletcher SP, Beran RK 2017 Identifying and characterizing interplay between hepatitis B virus X protein and Smc5/6. Viruses 9:E69. doi:10.3390/v9040069. 28368357 PMC5408675

[42] McNaughton AL, D’Arienzo V, Ansari MA, Lumley SF, Littlejohn M, Revill P, McKeating JA, Matthews PC 2019 Insights from deep sequencing of the HBV genome—unique, tiny, and misunderstood. Gastroenterology 156:384–399. doi:10.1053/j.gastro.2018.07.058. 30268787 PMC6347571

[43] Murakami S, Cheong JH, Kaneko S 1994 Human hepatitis virus X gene encodes a regulatory domain that represses transactivation of X protein. J Biol Chem 269:15118–15123. 8195148

[44] Misra KP, Mukherji A, Kumar V 2004 The conserved amino-terminal region (amino acids 1-20) of the hepatitis B virus X protein shows a transrepression function. Virus Res 105:157–165. doi:10.1016/j.virusres.2004.05.006. 15351489

[45] Altinel K, Hashimoto K, Wei Y, Neuveut C, Gupta I, Suzuki AM, Dos Santos A, Moreau P, Xia T, Kojima S, Kato S, Takikawa Y, Hidaka I, Shimizu M, Matsuura T, Tsubota A, Ikeda H, Nagoshi S, Suzuki H, Michel ML, Samuel D, Buendia MA, Faivre J, Carninci P 2016 Single-nucleotide resolution mapping of hepatitis B virus promoters in infected human livers and hepatocellular carcinoma. J Virol 90:10811–10822. doi:10.1128/JVI.01625-16. 27681123 PMC5110153

[46] Kwee L, Lucito R, Aufiero B, Schneider RJ 1992 Alternate translation initiation on hepatitis B virus X mRNA produces multiple polypeptides that differentially transactivate class II and III promoters. J Virol 66:4382–4389. 1318408 10.1128/jvi.66.7.4382-4389.1992 PMC241245

[47] Yan Y, Grant GA, Gross ML 2015 Hydrogen-deuterium exchange mass spectrometry reveals unique conformational and chemical transformations occurring upon [4Fe-4S] cluster binding in the type 2 L-serine dehydratase from Legionella pneumophila. Biochemistry 54:5322–5328. doi:10.1021/acs.biochem.5b00761. 26266572 PMC5993546

[48] Chalmers MJ, Busby SA, Pascal BD, Southern MR, Griffin PR 2007 A two-stage differential hydrogen deuterium exchange method for the rapid characterization of protein/ligand interactions. J Biomol Tech 18:194–204. 17916792 PMC2062560

[49] Pascal BD, Willis S, Lauer JL, Landgraf RR, West GM, Marciano D, Novick S, Goswami D, Chalmers MJ, Griffin PR 2012 HDX workbench: software for the analysis of H/D exchange MS data. J Am Soc Mass Spectrom 23:1512–1521. doi:10.1007/s13361-012-0419-6. 22692830 PMC3808162

[50] Zhang Z, Smith DL 1993 Determination of amide hydrogen exchange by mass spectrometry: a new tool for protein structure elucidation. Protein Sci 2:522–531. doi:10.1002/pro.5560020404. 8390883 PMC2142359

[51] Yang L, Adhikari J, Gross ML, Li L 2017 Kinetic isotope effects and hydrogen/deuterium exchange reveal large conformational changes during the catalysis of the Clostridium acetobutylicum spore photoproduct lyase. Photochem Photobiol 93:331–342. doi:10.1111/php.12697. 27992649 PMC5315627

[52] Cheng M, Zhang B, Cui W, Gross ML 2017 Laser-initiated radical trifluoromethylation of peptides and proteins: application to mass-spectrometry-based protein footprinting. Angew Chem Int Ed Engl 56:14007–14010. doi:10.1002/anie.201706697. 28901679 PMC5663992

[53] Hayer J, Jadeau F, Deleage G, Kay A, Zoulim F, Combet C 2013 HBVdb: a knowledge database for hepatitis B virus. Nucleic Acids Res 41:D566–D570. doi:10.1093/nar/gks1022. 23125365 PMC3531116

[54] Hall TA 1999 BioEdit: a user-friendly biological sequence alignment editor and analysis program for Windows 95/98/NT. Nucleic Acids Symp Ser (Oxf) 41:95–98.

