# Supplementary material for: Hepatitis B Virus X Protein Function Requires Zinc Binding
Source: J Virol. 2019 Jul 30;93(16):e00250-19. doi: 10.1128/JVI.00250-19 (PMC6675892; doi:10.1128/JVI.00250-19)
Supplement: Supplemental file 1 [file JVI.00250-19-s0001.pdf]

Supporting Information

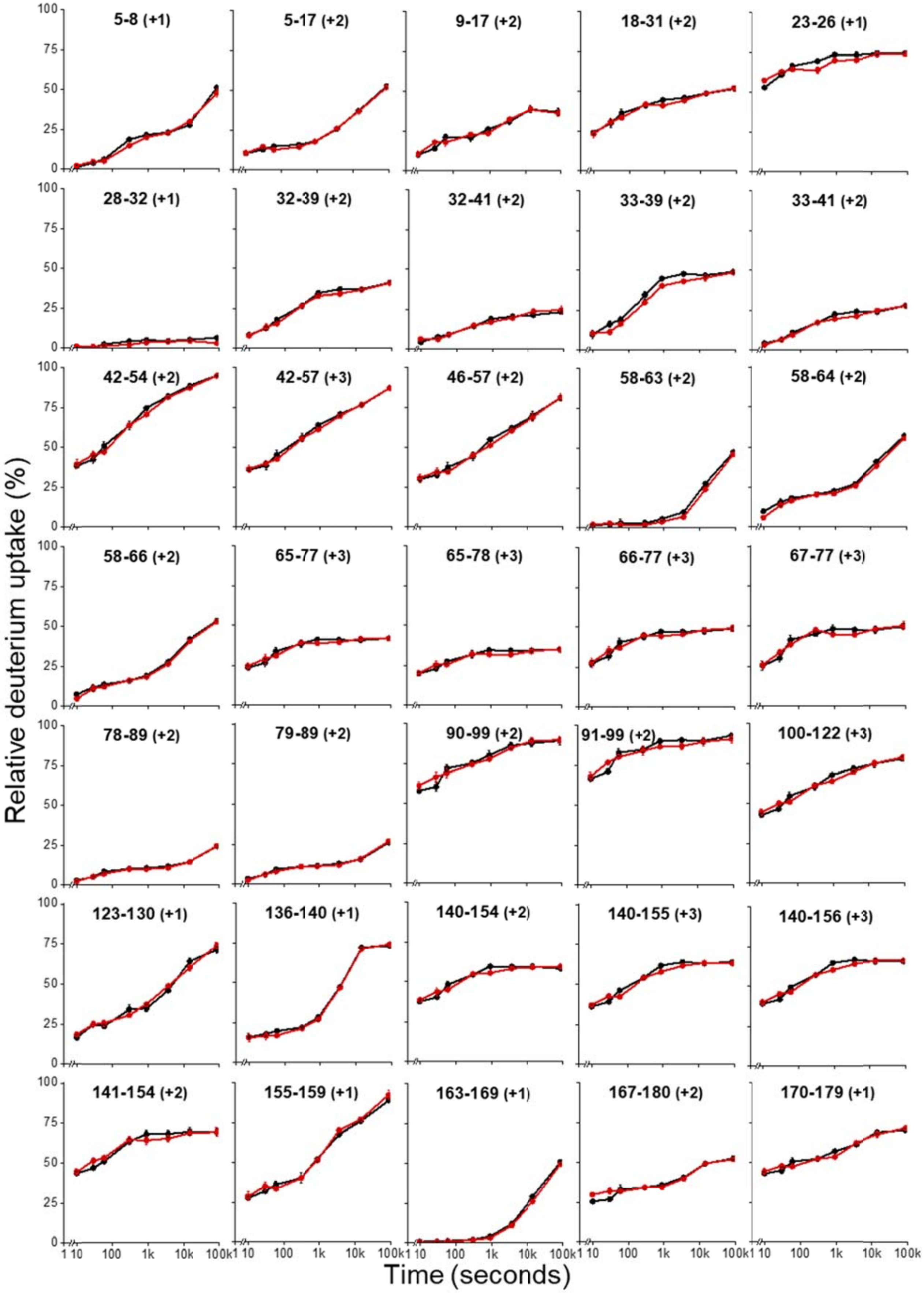

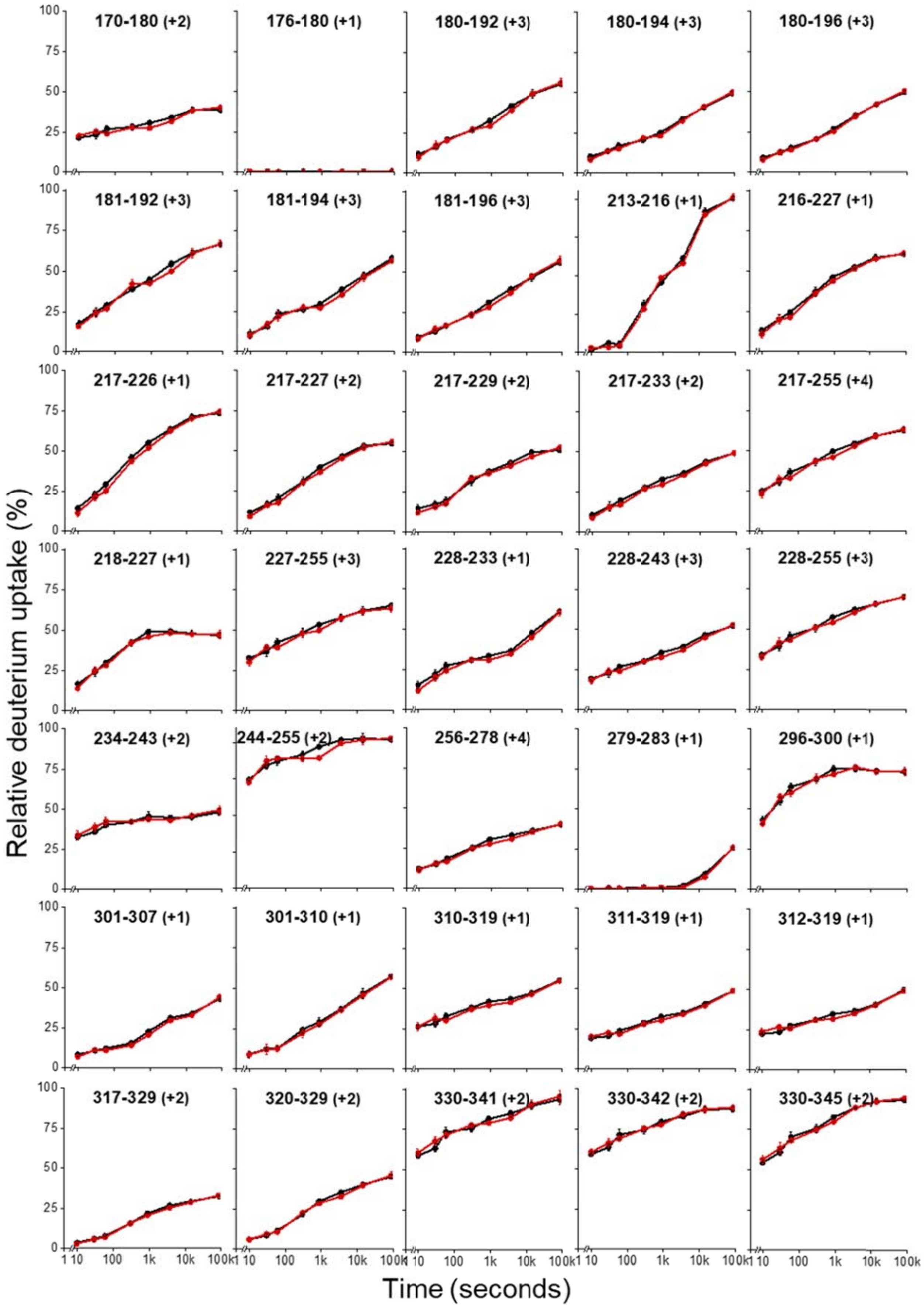
170-180 (+2)
176-180 (+1)
180-192 (+3)
180-194 (+3)
180-196 (+3)
181-192 (+3)
181-194 (+3)
181-196 (+3)
213-216 (+1)
216-227 (+1)
217-226 (+1)
217-227 (+2)
217-229 (+2)
217-233 (+2)
217-255 (+4)
218-227 (+1)
227-255 (+3)
228-233 (+1)
228-243 (+3)
228-255 (+3)
234-243 (+2)
244-255 (+2)
256-278 (+4)
279-283 (+1)
296-300 (+1)
301-307 (+1)
301-310 (+1)
310-319 (+1)
311-319 (+1)
312-319 (+1)
317-329 (+2)
320-329 (+2)
330-341 (+2)
330-342 (+2)
330-345 (+2)
Relative deuterium uptake (%)
Time (seconds)

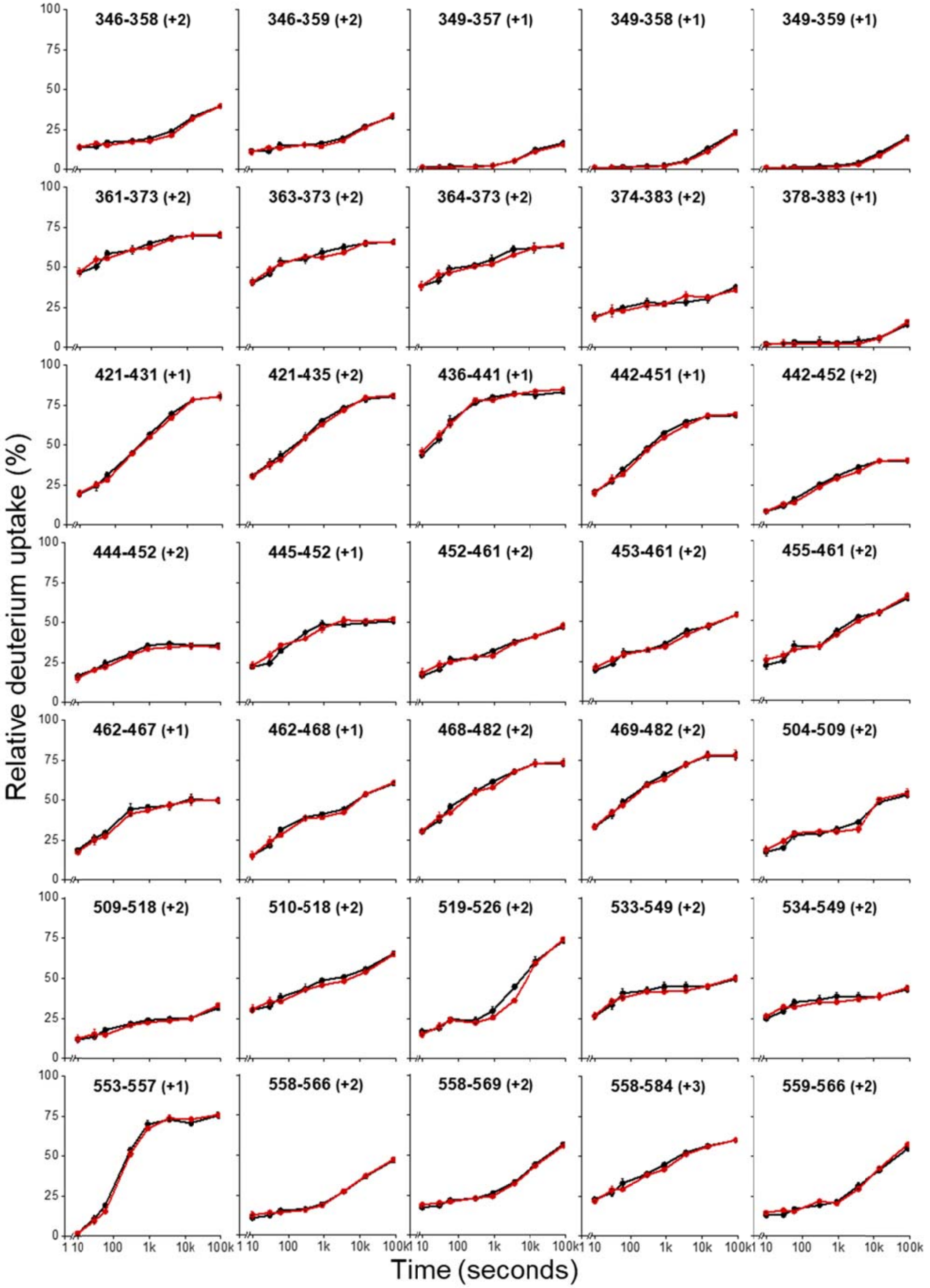
346-358 (+2)
346-359 (+2)
349-357 (+1)
349-358 (+1)
349-359 (+1)
361-373 (+2)
363-373 (+2)
364-373 (+2)
374-383 (+2)
378-383 (+1)
421-431 (+1)
421-435 (+2)
436-441 (+1)
442-451 (+1)
442-452 (+2)
444-452 (+2)
445-452 (+1)
452-461 (+2)
453-461 (+2)
455-461 (+2)
462-467 (+1)
462-468 (+1)
468-482 (+2)
469-482 (+2)
504-509 (+2)
509-518 (+2)
510-518 (+2)
519-526 (+2)
533-549 (+2)
534-549 (+2)
553-557 (+1)
558-566 (+2)
558-569 (+2)
558-584 (+3)
559-566 (+2)
Relative deuterium uptake (%)
Time (seconds)

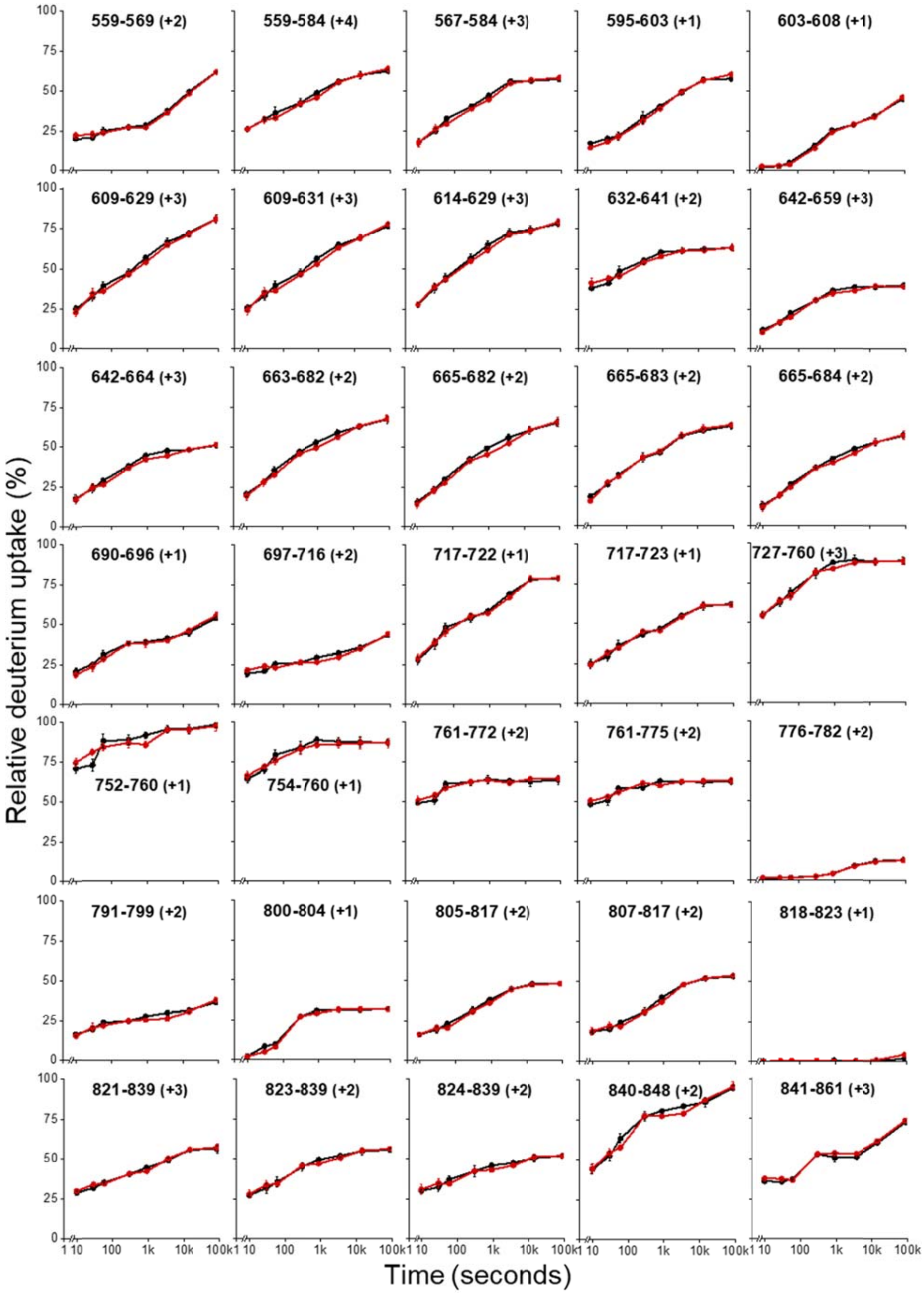

559-569 (+2)
559-584 (+4)
567-584 (+3)
595-603 (+1)
603-608 (+1)
609-629 (+3)
609-631 (+3)
614-629 (+3)
632-641 (+2)
642-659 (+3)
642-664 (+3)
663-682 (+2)
665-682 (+2)
665-683 (+2)
665-684 (+2)
690-696 (+1)
697-716 (+2)
717-722 (+1)
717-723 (+1)
727-760 (+3)
752-760 (+1)
754-760 (+1)
761-772 (+2)
761-775 (+2)
776-782 (+2)
791-799 (+2)
800-804 (+1)
805-817 (+2)
807-817 (+2)
818-823 (+1)
821-839 (+3)
823-839 (+2)
824-839 (+2)
840-848 (+2)
841-861 (+3)
Relative deuterium uptake (%)
Time (seconds)

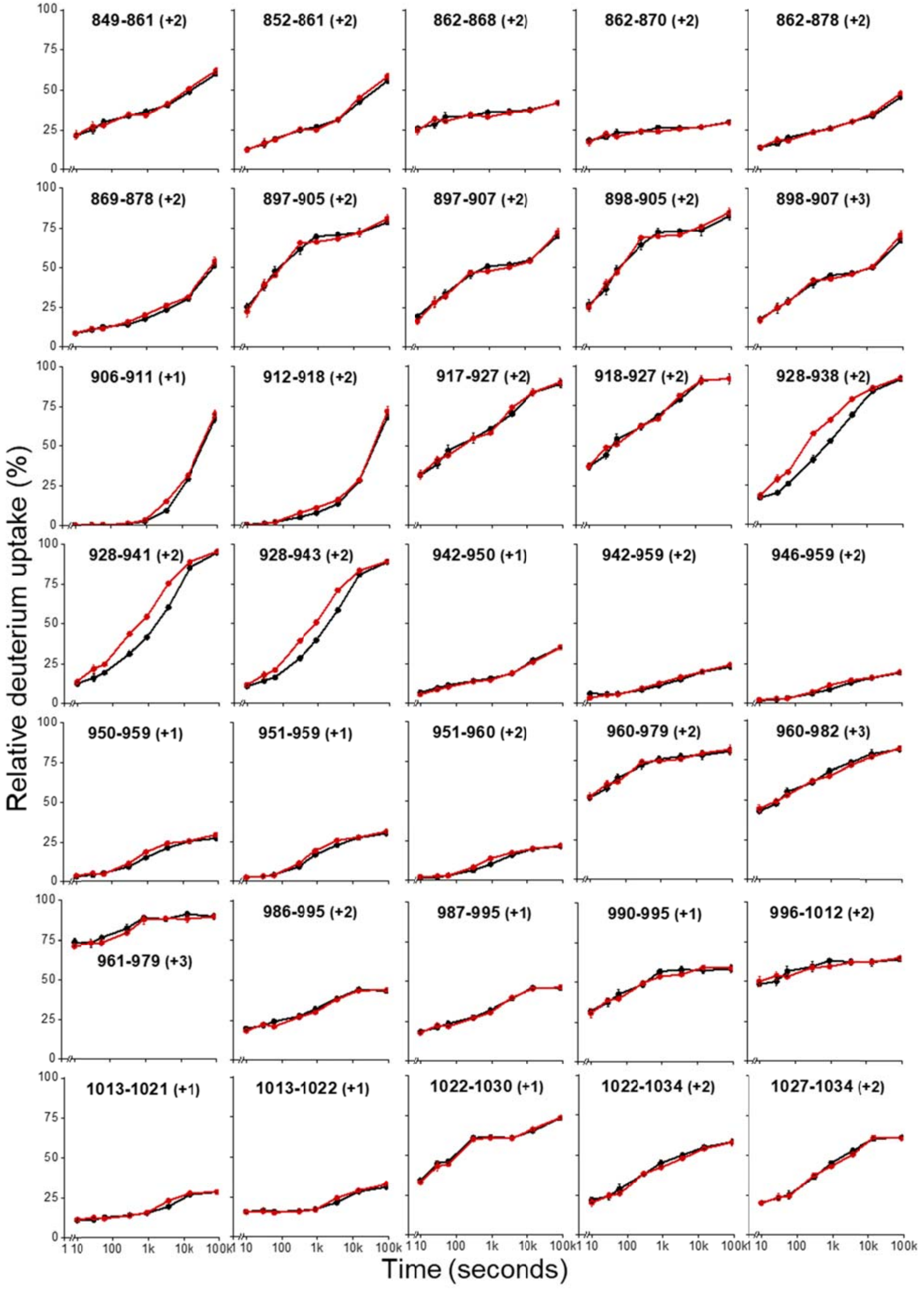
849-861 (+2)
852-861 (+2)
862-868 (+2)
862-870 (+2)
862-878 (+2)
869-878 (+2)
897-905 (+2)
897-907 (+2)
898-905 (+2)
898-907 (+3)
906-911 (+1)
912-918 (+2)
917-927 (+2)
918-927 (+2)
928-938 (+2)
928-941 (+2)
928-943 (+2)
942-950 (+1)
942-959 (+2)
946-959 (+2)
950-959 (+1)
951-959 (+1)
951-960 (+2)
960-979 (+2)
960-982 (+3)
961-979 (+3)
986-995 (+2)
987-995 (+1)
990-995 (+1)
996-1012 (+2)
1013-1021 (+1)
1013-1022 (+1)
1022-1030 (+1)
1022-1034 (+2)
1027-1034 (+2)
Relative deuterium uptake (%)
Time (seconds)

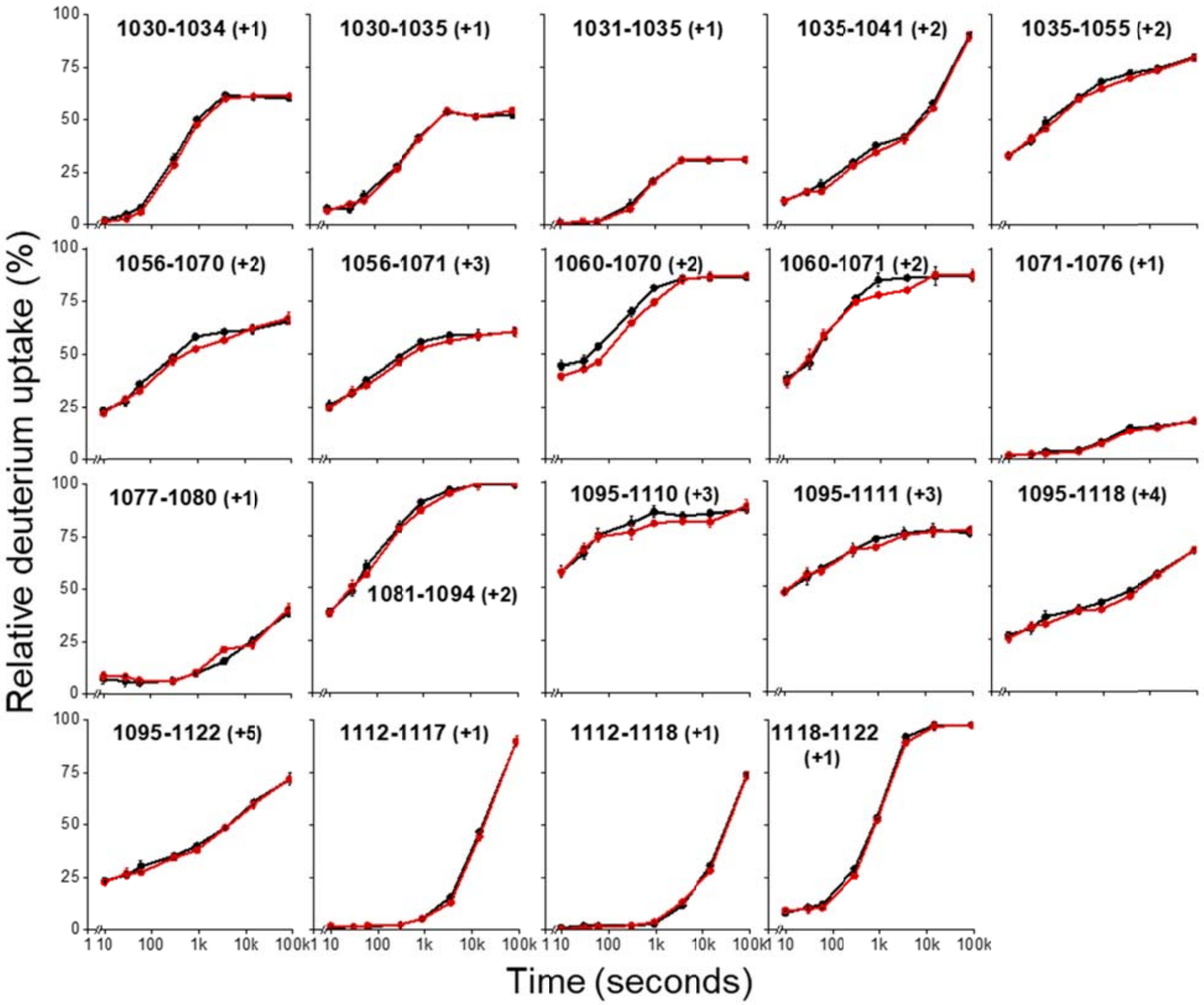

Supplementary Figure 1. HDX-MS analysis of DDB1 peptides in the co-expressed HBx:DDB1 complex. Comparison of deuterium uptake kinetic plots for all DDB1 peptides in the co-expressed HBx:DDB1 complex in the presence (black) and absence (red) of zinc. Circles indicate the mean and the error bars represent the standard deviation (SD). Amino acid sequence positions and the most abundant charge state for each peptide are displayed.

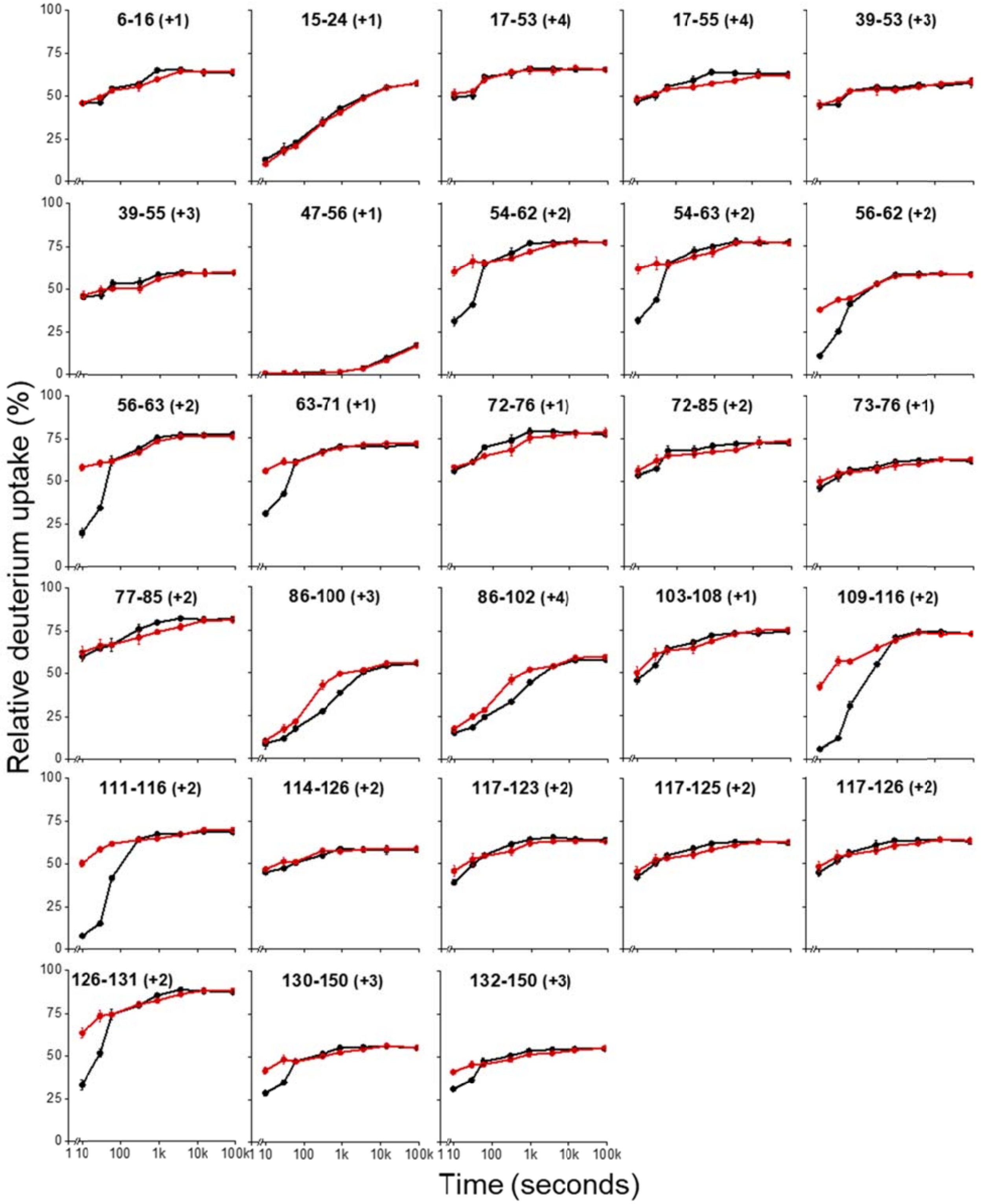

Supplementary Figure 2. HDX-MS analysis of HBx peptides in the co-expressed HBx:DDB1 complex. Comparison of deuterium uptake kinetic plots for all HBx peptides in the co-expressed HBx:DDB1 complex in the presence (black) and absence (red) of zinc. Circles indicate the mean and the error bars represent the SD. Amino acid sequence positions and the most abundant charge state for each peptide are displayed.

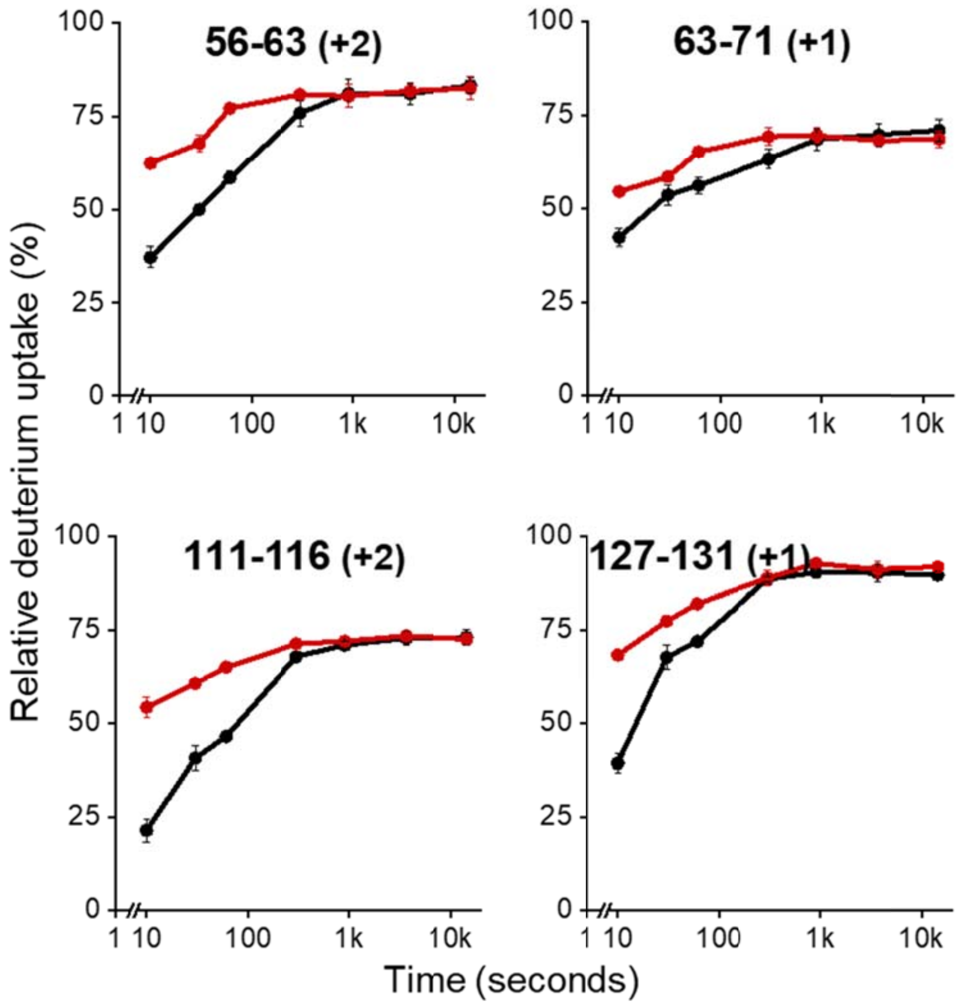

Supplementary Figure 3. HDX-MS analysis of HBx peptides in the HBx-DDB1 fusion protein. Comparison of deuterium uptake kinetic plots for select HBx peptides derived from representative regions of the HBx-DDB1 fusion protein in the presence (black) and absence (red) of zinc. The circles indicate the mean and the error bars represent the SD. Amino acid sequence positions and the most abundant charge state for each peptide are displayed. Only peptides which show a difference in deuterium uptake upon zinc removal are displayed.
